# Validity and reliability of inertial measurement units measurements for running kinematics in different foot strike pattern runners

**DOI:** 10.3389/fbioe.2022.1005496

**Published:** 2022-12-08

**Authors:** Ziwei Zeng, Yue Liu, Pan Li, Lin Wang

**Affiliations:** ^1^ School of Exercise and Health, Shanghai University of Sport, Shanghai, China; ^2^ Key Laboratory of Exercise and Health Sciences (Shanghai University of Sport), Ministry of Education, Shanghai, China

**Keywords:** validity, reliability, sensors, running, kinematic

## Abstract

This study aimed to assess the validity and reliability of the three-dimensional joint kinematic outcomes obtained by the inertial measurement units (IMUs) for runners with rearfoot strike pattern (RFS) and non-rearfoot strike pattern (NRFS). The IMUs system and optical motion capture system were used to simultaneous collect 3D kinematic of lower extremity joint data from participants running at 12 km/h. The joint angle waveforms showed a high correlation between the two systems after the offset correction in the sagittal plane (NRFS: coefficient of multiple correlation (CMC) = 0.924–0.968, root mean square error (RMSE) = 4.6^°^–13.7^°^; RFS: CMC = 0.930–0.965, RMSE = 3.1^°^–7.7^°^), but revealed high variability in the frontal and transverse planes (NRFS: CMC = 0.924–0.968, RMSE = 4.6°–13.7°; RFS: CMC = 0.930–0.965, RMSE = 3.1°–7.7°). The between-rater and between-day reliability were shown to be very good to excellent in the sagittal plane (between-rater: NRFS: CMC = 0.967–0.975, RMSE = 1.9°–2.9°, RFS: CMC = 0.922–0.989, RMSE = 1.0°–2.5°; between-day: NRFS: CMC = 0.950–0.978, RMSE = 1.6°–2.7°, RFS: CMC = 0.920–0.989, RMSE = 1.7°–2.2°), whereas the reliability was weak to very good (between-rater: NRFS: CMC = 0.480–0.947, RMSE = 1.1°–2.7°, RFS: CMC = 0.646–0.873, RMSE = 0.7°–2.4°; between-day: NRFS: CMC = 0.666–0.867, RMSE = 0.7°–2.8°, RFS: CMC = 0.321–0.805, RMSE = 0.9°–5.0°) in the frontal and transverse planes across all joints in both types of runners. The IMUs system was a feasible tool for measuring lower extremity joint kinematics in the sagittal plane during running, especially for RFS runners. However, the joint kinematics data in frontal and transverse planes derived by the IMUs system need to be used with caution.

## Introduction

Running has been related with health benefits, but it also may lead to running-related sports injuries (RRIs) (Arnold and Moody, 2018). The incidence of RRIs is approximately 19.4%–79.3% (van Gent et al., 2007), with a risk of 2.5–33 sports injuries per 1,000 h of running (Videbaek et al., 2015). Considering the high prevalence and incidence, biomechanical variables have been investigated by using various instrumented motion analysis systems to reveal the specific biomechanical mechanisms underlying the occurrence of RRIs (Caldas et al., 2017; Camomilla et al., 2017; Petraglia et al., 2019). The inability to adapt to repetitive stresses during running puts the body in a state of overload and triggers RRIs (Hreljac et al., 2000), and the abnormal running kinematic pattern is one of the major risk factors for causing RRIs (Thompson et al., 2022). Thus, running kinematic data obtained through instrumented motion analysis systems are of practical importance for assessing motion performance and preventing the RRIs occurrence (Cheung et al., 2017).

Three-dimensional motion capture systems are the gold standard for instrumented motion analysis (Camomilla et al., 2017; Petraglia et al., 2019). However, environmental constraints, the need for professional operation, cumbersome testing procedures, high prices and poor portability have led to many inconveniences in their practical application (Benson et al., 2019). As alternative devices with the advantages of portability, cost effectiveness and ease of operation, inertial sensors have recently become increasingly popular in sports motion measurements and widely used in laboratory and outdoor approach (Ciuti et al., 2015; Caldas et al., 2017; Poitras et al., 2019; Vienne-Jumeau et al., 2019). With the development of technology, inertial sensors have evolved from a single accelerometer to a combination of accelerometers, gyroscopes and/or magnetometers known as inertial measurement units (IMUs) or micro-electro-mechanical systems (MEMS) (Filippeschi et al., 2017). IMUs can capture linear acceleration, angular velocity and direction during motion in real time, enabling timely kinematic measurements through data integration (Kok and Schön, 2016; Al-Amri et al., 2018; Mavor et al., 2020).

To provide evidential support for the practical application of IMUs in running, previous studies have extensively investigated gait spatiotemporal parameters obtained from IMUs and optical reference systems to evaluate the validity and reliability of IMUs (Bergamini et al., 2012; Koldenhoven and Hertel, 2018). Most of them, such as step length, step frequency and cycle time, exhibited good validity and reliability (Bergamini et al., 2012; Koldenhoven and Hertel, 2018; Zeng et al., 2022). It was confirmed that shoe type, running speed, IMUs position and gait strategy can affect the IMU measurements (Gilgen-Ammann et al., 2017; Rabuffetti et al., 2019; Napier et al., 2021).

As we know, different foot strike patterns (FSPs) were performed in human running (Daoud et al., 2012; Knorz et al., 2017). FSP as an important external factor influencing running gait (Wei et al., 2019), yet studies exploring whether FSP affects the validity and reliability of IMUs measurements are still lacking. According to the initial contact between the foot and the ground during running, FSPs fall into three categories, namely, forefoot strike pattern (FFS), mid-foot strike pattern (MFS) and rearfoot strike pattern (RFS) (Lieberman et al., 2010). In an Asian population-based long-distance running race, FFS, MFS and RFS runners represent 1.7%, 16.6%, and 71.1% of the runners, respectively (Patoz et al., 2019). Considering the relatively small number of runners landing on FFS and MFS, they were mostly co-classified as non-RFS (NRFS) for research (Wei et al., 2020). As the farthest segment of the human body, the feet come in contact with the ground and propel the body forward during running (Suwa et al., 2017). Different FSPs will change the stress patterns and present biomechanical differences, thus triggering varied RRIs (Daoud et al., 2012; Knorz et al., 2017). This phenomenon is not only manifested in the sagittal plane, but also in the frontal and transversal planes for runners with different FSPs, which may be the reason why runners with different FSPs tend to develop RRIs in different parts of the body (De Wit et al., 2000; Hamill and Gruber., 2017; Patoz et al., 2019; Ogasawara et al., 2021).

In previous studies, the validity and reliability of IMUs measurements for running kinematics have been well investigated in the sagittal plane (Nuesch et al., 2017; de Ruiter et al., 2022). To our knowledge, there is a lack of comprehensive studies to investigate whether FSP affects the validity and reliability of 3D lower extremity kinematics measured by IMUs. Therefore, this study aimed to assess the validity and reliability of the 3D joint kinematic outcomes obtained by the IMUs for runners with different FSPs, which were collected simultaneously by the IMUs and the optical motion capture system during running. We hypothesised that joint angle waveforms and the discrete parameters derived from the IMUs system had high validity and reliability in both patterns of runners in the sagittal plane, but these were poorer in the frontal and transverse planes.

## Materials and methods

### Participants

Sample size estimation revealed that 24 participants were required to detect an expected difference in the validity and reliability of IMUs in healthy adult with an effect size of at least 0.5 with 0.8 power at a 5% significance level (Kobsar et al., 2020). Thus, 15 habitual recreational male RFS runners and fifteen age matched habitual recreational male NRFS runners were recruited from the local college. Runners are classified according to United States Track & Field criteria based on age, gender and recent race performance (USA Track and Field, 2016). Specifically, runners who had never competed or had recently participated in a middle-to long-distance race and achieved an age-graded score < 60% were called recreational runners (Clermont et al., 2019). All participants were right-leg dominant; this was the preferred leg for kicking a ball (Ghena et al., 1991).

**FIGURE 1:**
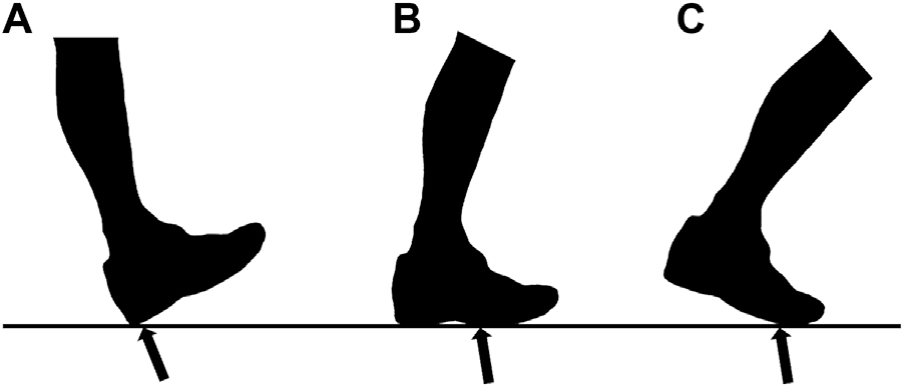
Three common types of foot strike pattern while running. **(A)** rearfoot strike pattern; **(B)** mid-foot strike pattern; **(C)** forefoot strike pattern.

Upon arrival at the laboratory, participants completed a verbal questionnaire to ensure that they had no known abnormalities or recent musculoskeletal injuries (within the past 6 months) that could affect their running performance. They reported their own FSP. Confirm the foot landing pattern according to the primary contact area between the foot and the ground in the running process, divided into RFS and NRFS (Lieberman et al., 2010) (Figure 1). Before the formal test, the participants’ body height, body mass and leg length were measured by rater 1 (ZZ). Participants were instructed to run at their preferred speed on the treadmill in their habitual landing patterns, two assessors [rater 1 and rater 2 (YL)] determined the habitual FSP of the participants by measuring the foot strike angle. The angle was defined as the angle of the foot when it touches the ground minus the angle when it stands (Altman and Davis, 2012). A positive angle indicated RFS and a negative angle indicated NRFS (Forrester & Townend, 2015). This study was approved by the Ethics Committee of Shanghai University of Sport (the registration number: 102772021RT129). Written informed consent was obtained from each participant before data collection.

### Testing equipment and procedure

Experiments were conducted at the biomechanics laboratory in Shanghai University of Sport. All participants wore standardised running shoes (ASICS, SORTIEMAGIC RP 4 TMM467.0790, Japan) in their preferred shoe size. Data were collected simultaneously by the IMUs system and optical motion capture system through an external synchronisation system.

The IMUs system (STT system, Basque Country, Spain) contains seven IMUs, each consisting of a triaxial accelerometer (± 16 g), gyroscope (± 1,200°/s) and magnetometer (± 1.3 Gs) with onboard processing and memory capabilities to capture kinematic data at a sampling frequency of 200 Hz. The IMUs were placed on the participant’s sacrum in the mid-line of the back around the S1 level and bilaterally on the anterior midthigh, anterior upper shank and dorsum of the foot according to the IMUs measurement guidelines (Figure 1). Calibration of IMUs was completed by participants standing stationary for 5 s. Hip adduction and internal rotation, knee adduction and internal rotation and ankle inversion were defined as negative angles. Thus, they were multiplied by −1 to be aligned with the corresponding angles of the optical reference system.

**FIGURE 2:**
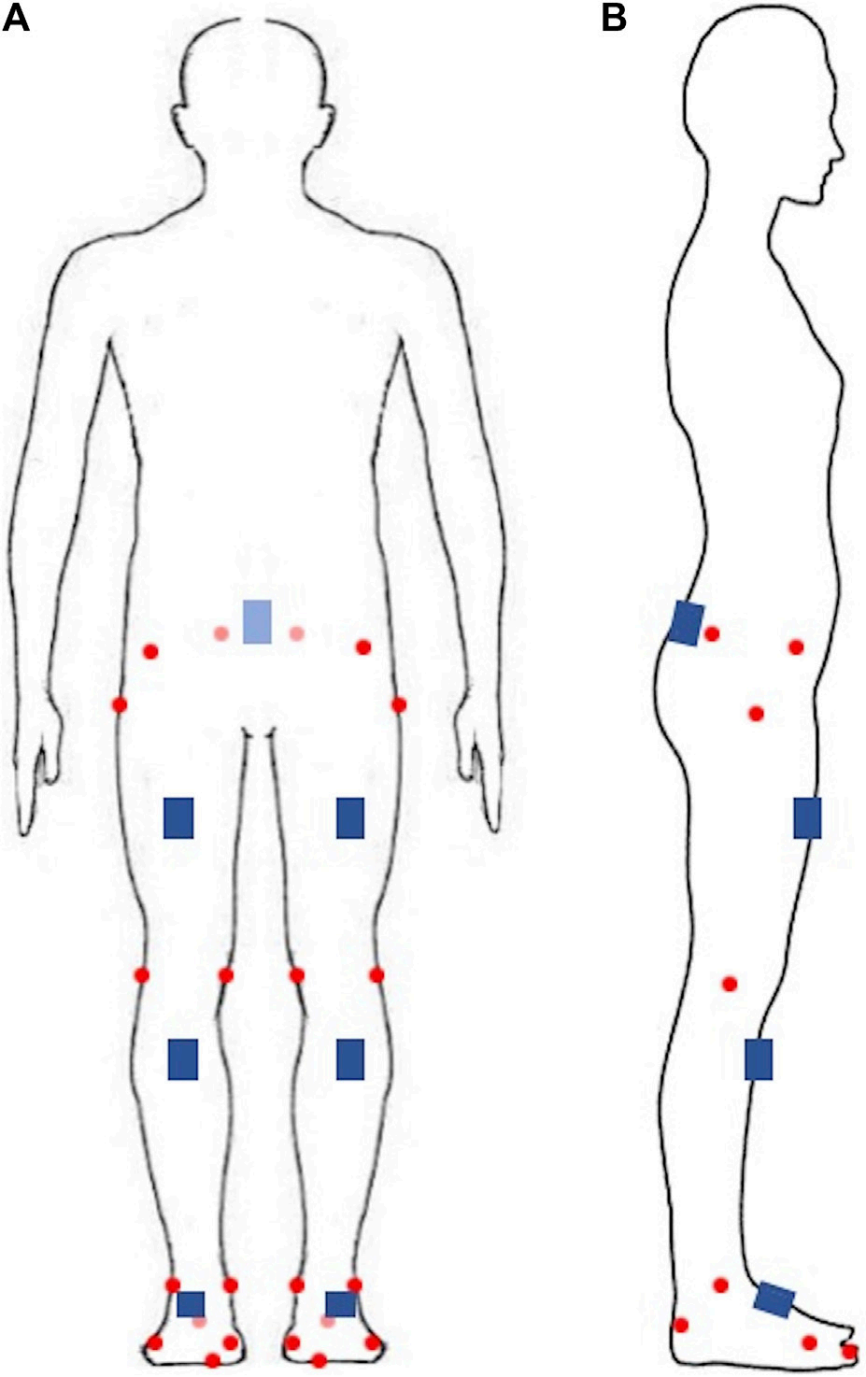
The retroreflective markers and inertial measurement units’ attachment site. **(A)** front view; **(B)** side view. Red circles represent retroreflective markers, blue squares represent inertial measurement units.

Twenty-two retroreflective markers were placed bilaterally on the participant’s anterior superior iliac spine, posterior superior iliac spine, greater tubercle, medial epicondyle of femur, lateral epicondyle of femur, medial malleoli, lateral malleoli, the first and fifth metatarsal heads and the anterior and posterior tip of shoe based on gait-in-plug (Figure 2). Before the running test, a static calibration trial in neutral upright position was recorded for subsequent analysis in the Nexus and Visual 3D software. Similarly, knee flexion was defined as a negative angle. Hence, it was multiplied by −1 to match the angle of the IMUs system. The optical reference system consists of a motion capture system (Vicon T40, Oxford Metrology, United Kingdom) with a sampling frequency of 200 Hz from eight high speed cameras and a dual-belt instrumented treadmill (Bertec, Columbus, OH, United States) with a sampling frequency of 1,000 Hz for simultaneous acquisition of markers’ trajectory and ground reaction force during running.

The experiment included three sessions in 2 days. Before the beginning of each day’s formal running trial, retroreflective markers were attached to the participant’s body surface by the same experienced researcher. Then, the participant completed a 5 min warm-up, that is, to run on the treadmill at self-selected speed. In the first session, rater 1 used nylon straps to attach the IMUs to the corresponding site and participants completed 2 min running tests at the speed of 12 km/h. After that, rater 1 removed the IMUs. Participants were allowed to rest for 10 min to avoid fatigue, which affected the measurement results (Konharn et al., 2016). In the second session, the IMUs were attached by rater 2. Participants completed the running test. After that, rater 2 removed the IMUs. Session 3 was conducted at the same time 7 days later, while participants completed the same running test with the IMUs attached and removed by rater 1. The IMUs may shift their position with the movement. The rater corresponding to each session was responsible for adjusting the IMUs position before each running trial.

### Data processing

In each test session, the same running gait cycles measured by the two systems were processed by confirming the synchronisation signal. Ten consecutive gait cycles in the middle time phase of running were selected and only the right limb was analyzed. The gait cycle was normalised by time and defined as starting from the foot initial contact the ground and to the end of the next contact. All outcomes were represented by the ensemble mean of the 10 gait cycles.

In the Vicon system, the 3D kinematic data of the hip, knee and ankle joints in the gait cycle were calculated using Visual 3D software. A ground reaction force cutoff value of 50 N was used to identify foot strike and toe-off events (Napier et al., 2021). In the STT system, the joint angles over time were exported as CSV files along with the times corresponding to the foot strike and toe-off events. The times from foot strike to the next foot strike, from foot strike to toe-off and from toe-off to the next foot strike were defined as the gait stride, the stance phase and the swing phase, respectively. The 90 discrete parameters calculated for the lower limb joints in the sagittal, frontal and transverse planes over 10 gait cycles for the two measurement systems were calculated, including the following: hip, knee and ankle angles at foot strike; maximum hip, knee and ankle angles during gait stride, stance phase and swing phase; minimal hip, knee and ankle angles during gait stride, stance phase and swing phase; and hip, knee and ankle range of motion (ROM) during gait stride, stance phase and swing phase (Nuesch et al., 2017). For the Vicon system, these parameters are obtained by analysis in the Visual 3D software through a pre-programmed procedure; for the sensor system, since the joint angle data can be directly exported, the joint angles corresponding to these specific time points need to be confirmed by matching the time points of the gait events exported by the STT system, the ROM was obtained by subtracting the minimal angle from the maximum angle in the Microsoft Excel.

### Statistical analysis

Statistical data were performed in Matlab (Version 2015a; MathWorks Inc., Natick, MA, United States) and SPSS (Version 26, IBM SPSS Inc., Chicago, United States). Descriptive statistics (mean ± SD) were used to report the demographic data of the participants. All data were normally distributed on the basis of Shapiro-Wilk test. The concurrent validity, between-rater reliability and between-day reliability of the IMUs measurements were evaluated separately in two aspects, namely, the joint angle waveform and the discrete parameters.

The concurrent validity was evaluated using data from session 1 conducted by rater 1. For the joint angle waveform, coefficient of multiple correlation (CMC) and root mean square error (RMSE) were calculated for each condition, which have been wildly used to evaluate the consistency of the waveforms (Ferrari et al., 2010a; Ferrari et al., 2010b; Nuesch et al., 2017). CMC is a metric that measures the overall similarity of the waveforms and calculated before and after the offset correction between the waveforms of the two systems, considering the concurrent effects of differences in offset, correlation and gain (Ferrari et al., 2010a; Roislien et al., 2012; Nuesch et al., 2017; Al-Amri et al., 2018), where CMC < 0.65 indicates weak, 0.65 ≤ CMC < 0.75 indicates moderate, 0.75 ≤ CMC < 0.85 indicates good, 0.85 ≤ CMC < 0.95 indicates very good and CMC ≥ 0.95 indicates excellent (Ferrari et al., 2010b). When the variability between joint angle waveforms was lower, the CMC was closer to 1. Otherwise, the CMC tended to be zero or even became a complex number (Ferrari et al., 2010a). Spearman’s correlation coefficient (r) and RMSE were calculated for each discrete parameter. Correlation coefficients were interpreted as poor (0–0.49), moderate (0.50–0.74) and strong (0.75–1) (Shrout & Fleiss, 1979). After removing the offset between the joint angle waveforms between the two system for each participant, the above analyses were repeated.

Between-rater (within-session) reliability was evaluated by comparing data from session 1 (rater 1) and session 2 (rater 2). Between-day (within-rater) reliability was evaluated by comparing data from sessions 1 and 2. The reliability of the joint angle waveform was also evaluated using CMC and RMSE. The intra-class correlation coefficients (ICCs) with their 95% confidence intervals (95% CI) were calculated using a two-way, random single measure analysis and RMSE were used to estimate the reliability of discrete parameters for each condition. Point estimates of the ICCs were rated as poor (0–0.39), moderate (0.4–0.73), good (0.74–0.9) and excellent (0.9–1) (Fleiss, 1999). All the *p*-values < 0.05 of statistical tests were considered to be statistically significant.

## Results

### Demographics

The data of 15 recreational male RFS runners and 15 recreational male NRFS runners were analysed. Characteristics of the participants are presented in Table 1.

**TABLE 1:** Characteristics of participants.

	NRFS (*n* = 15)	RFS (*n* = 15)	*p*
Age (years)	22.1 ± 2.0	20.8 ± 1.8	0.080
Height (cm)	177.4 ± 5.4	177.7 ± 6.5	0.904
Mass (kg)	67.4 ± 4.7	68.3 ± 8.3	0.742
BMI (kg/m^2^)	21.5 ± 1.5	21.6 ± 1.7	0.842
Leg length (cm)	90.6 ± 3.6	92.5 ± 5.0	0.260
Running age (years)	4.5 ± 2.2	5.5 ± 3.0	0.317
Weekly running mileage (km)	24.6 ± 21.9	23.6 ± 23.6	0.905

NRFS, non-rearfoot strike pattern; RFS, rearfoot strike pattern; BMI, body mass index.

### Validity

#### Joint angle waveforms

**FIGURE 3:**
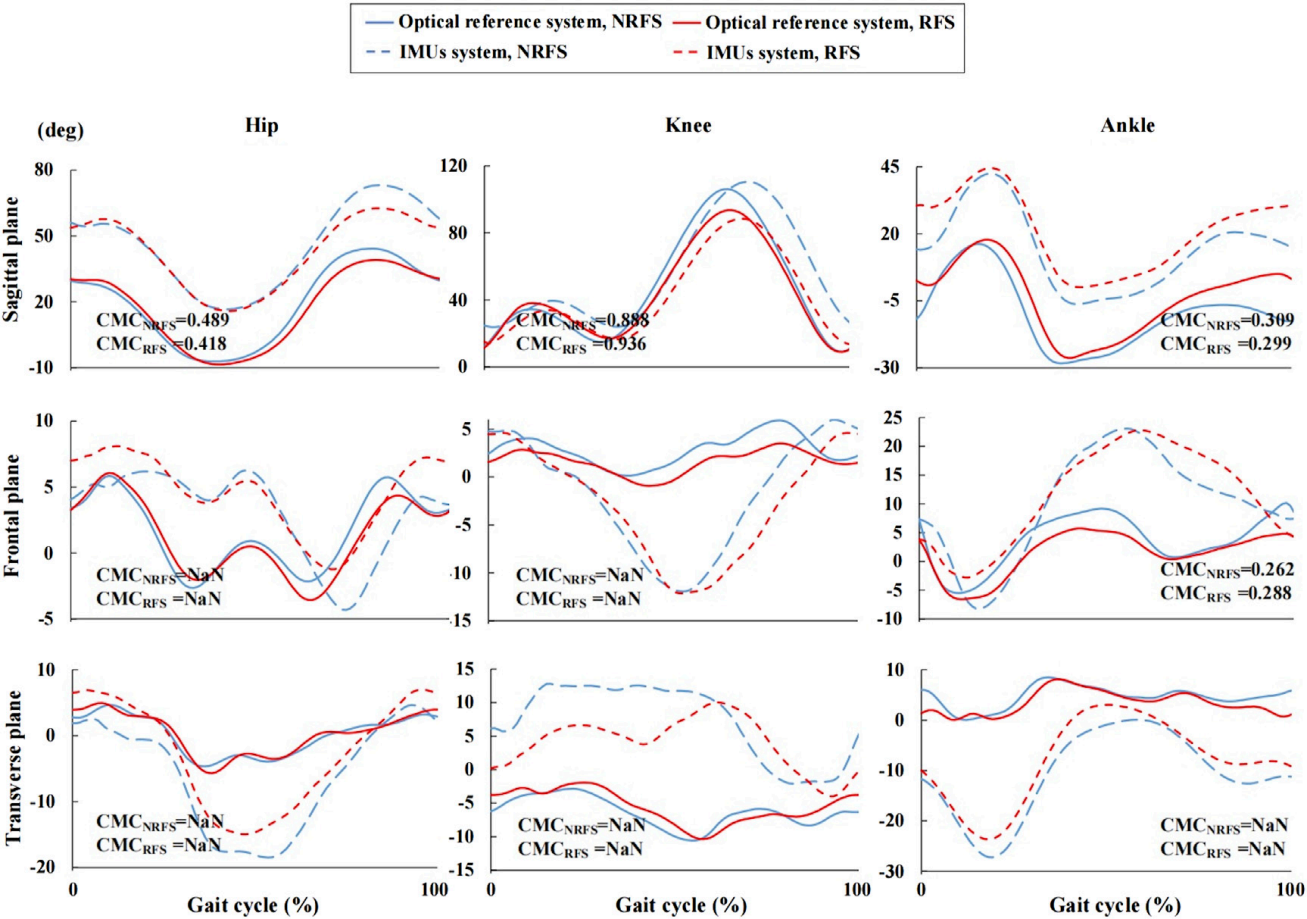
Joint angle waveforms during gait cycle measured by the inertial measurement units and optical reference system before offset correction.

**FIGURE 4:**
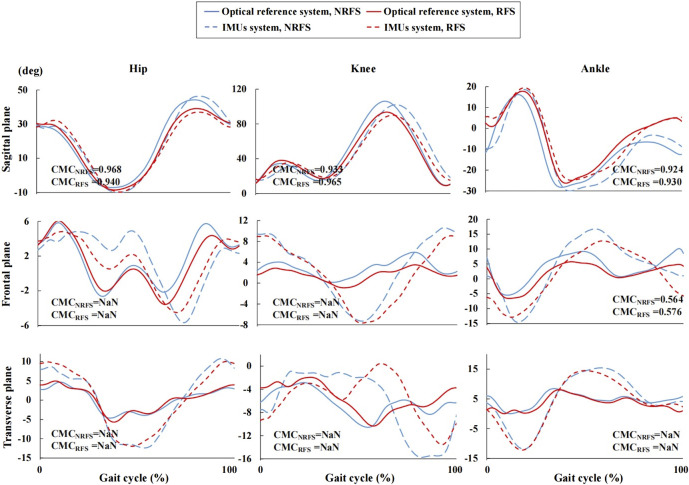
Joint angle waveforms during gait cycle measured by the inertial measurement units and optical reference system after offset correction. NRFS non-rearfoot strike pattern; RFS rearfoot strike pattern; IMUs inertial measurement units; CMC_NRFS_ the coefficient of multiple correlation of the two systems in NRFS runners; CMC_RFS_ the coefficient of multiple correlation of the two systems in RFS runners; NaN indicates that the CMC is a complex number.

Figure 3 clearly shows the vertical shift in the joint angle waveforms in the sagittal plane between the two systems. Before the offset correction, very good validity was shown for the knee joint waveform (NRFS: CMC = 0.888; RFS: CMC = 0.936) measured by the IMUs, whereas only weak validity was shown for the hip (NRFS: CMC = 0.489; RFS: CMC = 0.418) and ankle (NRFS: CMC = 0.309; RFS: CMC = 0.299) joint angle waveforms in both types of runners in the sagittal plane. Removed offset resulted in very good to excellent CMC for all joints in the sagittal plane (NRFS: CMC = 0.924–0.968; RFS: CMC = 0.930–0.965) (Figure 4).

The ankle joint angle waveforms in the frontal plane showed weak correlation between the two systems (before offset correction: NRFS: CMC = 0.262; RFS: CMC = 0.288; after offset correction: NRFS: CMC = 0.564; RFS: CMC = 0.576), In both frontal and transverse planes, the CMC values of other joint angle waveforms between the two systems were complex number, which indicates that there was obvious variability between the two systems both before and after offset correction (Figures 3, 4).

**FIGURE 5:**
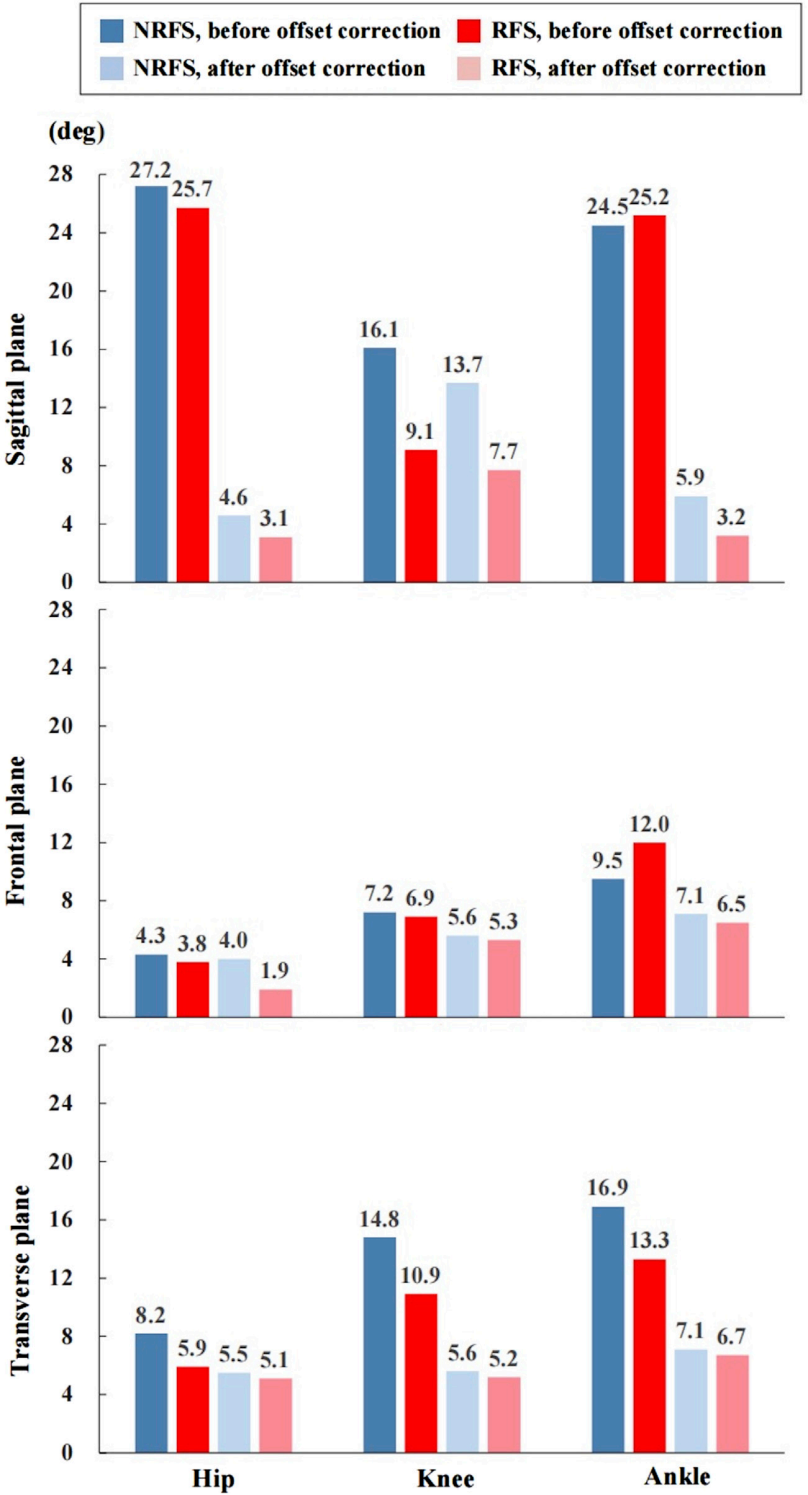
The root mean square error of the joint angle waveforms between the inertial measurement units system and optical reference system. NRFS non-rearfoot strike pattern; RFS rearfoot strike pattern.

The RMSE of all joint angle waveforms in the sagittal plane before the offset correction were in the ranges of 16.1°–27.2° and 9.1°–25.7° in NRFS and RFS runners; after the offset correction, they were in the ranges of 4.6°–13.7° and 3.1°–7.7°, respectively (Figure 5).

The RMSE of all joint angle waveforms in the frontal plane before the offset correction were in the ranges of 4.3°–9.5° and 3.8°–12.0° in NRFS and RFS runners; after the offset correction, they were in the ranges of 4.0°–7.1° and 1.9°–6.5°, respectively (Figure 5).

The RMSE of all joint angle waveforms in the transverse plane before the offset correction were in the ranges of 8.2°–16.9° and 5.9°–13.3° in NRFS and RFS runners; after the offset correction, they were in the ranges of 5.5°–7.1° and 5.1°–6.7°, respectively (Figure 5).

### Discrete parameters

#### Correlation

Before the offset correction, except for the touchdown angle of the knee joint in the transverse plane for RFS runners (r = 0.789, *p* < 0.001), few parameters in the sagittal plane showed strong correlation between the two systems, such as hip and knee ROM in the cycle and swing phase (r ≥ 0.829, *p* < 0.001) and ankle ROM in the swing phase (r = 0.939, *p* < 0.001) in NRFS runners; ankle minimum angle in the cycle and swing phase and ROM in the cycle (r ≥ 0.850, *p* < 0.001) in RFS runners. For all other discrete parameters, only poor to moderate correlations were shown between the two systems (Supplementary Table S1).

After the offset correction, for the hip joint, the correlations between the two systems in the NRFS runners for the discrete parameters other than ROM were in the ranges of 0.693–0.957, 0.136–0.496, and 0.143–0.893 in the sagittal, frontal and transverse planes, respectively; for the RFS runners, the correlations between the two systems were in the ranges of 0.518–0.900, 0.471–0.854, and 0.579–0.954 in the sagittal, frontal and transverse planes, respectively. For the knee joint, the correlations between the two systems in the NRFS runners for the discrete parameters other than ROM were in the ranges of 0.493–0.971, 0.361–0.796, and 0.218–0.614 in the sagittal, frontal and transverse planes, respectively; for the RFS runners, the correlations between the two systems were in the ranges of 0.696–0.882, 0.139–0.632, and 0.211–0.904 in the sagittal, frontal and transverse planes, respectively. For the ankle joint, the correlations between the two systems in the NRFS runners for the discrete parameters other than ROM were in the ranges of −0.150–0.954, −0.393–0.796, and −0.154–0.696 in the sagittal, frontal and transverse planes, respectively; for the RFS runners, the correlations between the two systems were in the ranges of 0.248–0.965, 0.055–0.589, and 0.371–0.796 in the sagittal, frontal and transverse planes, respectively (Supplementary Table S1).

### RMSE

#### Hip

Supplementary Table S1 presents results from the RMSE of discrete parameters between the two systems before and after the offset correction. Before the offset correction, in the sagittal plane, the calculated RMSEs were in the ranges of 6.5°–31.5° and 7.6°–30.6° in the NRFS and RFS runners, respectively; in the frontal plane, the calculated RMSEs were in the ranges of 5.4°–9.5° and 4.1°–8.0° in the NRFS and RFS runners, respectively; in the transverse plane, the calculated RMSE were in the ranges of 9.0°–17.3° and 7.8°–14.9° in the NRFS and RFS runners, respectively.

After the offset correction, in the sagittal plane, the calculated RMSEs were in the ranges of 3.2°–6.5° and 3.2°–6.6° in the NRFS and RFS runners, respectively; in the frontal plane, the calculated RMSEs were in the ranges of 2.6°–4.2° and 2.0°–3.0° in the NRFS and RFS runners, respectively; in the transverse plane, the calculated RMSE were in the ranges of 5.2°–9.0° and 5.7°–8.8° in the NRFS and RFS runners, respectively (Supplementary Table S1).

#### Knee

Supplementary Table S1 presents results from the RMSE of discrete parameters between the two systems before and after the offset correction. Before the offset correction, in the sagittal plane, the calculated RMSEs were in the ranges of 4.6°–18.0° and 8.1°–13.8° in the NRFS and RFS runners, respectively; in the frontal plane, the calculated RMSE were in the ranges of 7.6°–17.0° and 5.6°–16.2° in the NRFS and RFS runners, respectively; in the transverse plane, the calculated RMSE were in the ranges of 9.0°–24.8° and 2.9°–16.3° in the NRFS and RFS runners, respectively.

After the offset correction, in the sagittal plane, the calculated RMSEs were in the ranges of 3.2°–7.0° and 3.1°–8.7° in the NRFS and RFS runners, respectively; in the frontal plane, the calculated RMSEs were in the ranges of 5.2°–9.7° and 3.7°–9.5° in the NRFS and RFS runners, respectively; in the transverse plane, the calculated RMSE were in the ranges of 8.1°–14.0° and 2.4°–8.5° in the NRFS and RFS runners, respectively (Supplementary Table S1).

#### Ankle

Supplementary Table S1 presents results from the RMSE of discrete parameters between the two systems before and after the offset correction. Before the offset correction, in the sagittal plane, the calculated RMSEs were in the ranges of 3.9°–28.4° and 5.7°–30.1° in the NRFS and RFS runners, respectively; in the frontal plane, the calculated RMSE were in the ranges of 4.0°–24.8° and 8.5°–22.7° in the NRFS and RFS runners, respectively; in the transverse plane, the calculated RMSE were in the ranges of 15.7°–30.1° and 7.4°–25.2° in the NRFS and RFS runners, respectively.

After the offset correction, in the sagittal plane, the calculated RMSEs were in the ranges of 1.9°–14.2° and 2.3°–18.3° in the NRFS and RFS runners, respectively; in the frontal plane, the calculated RMSEs were in the ranges of 6.9°–15.3° and 6.6°–15.2° in the NRFS and RFS runners, respectively; in the transverse plane, the calculated RMSE were in the ranges of 3.9°–14.3° and 3.1°–13.4° in the NRFS and RFS runners, respectively (Supplementary Table S1).

## Reliability

### Joint angle waveforms

**FIGURE 6:**
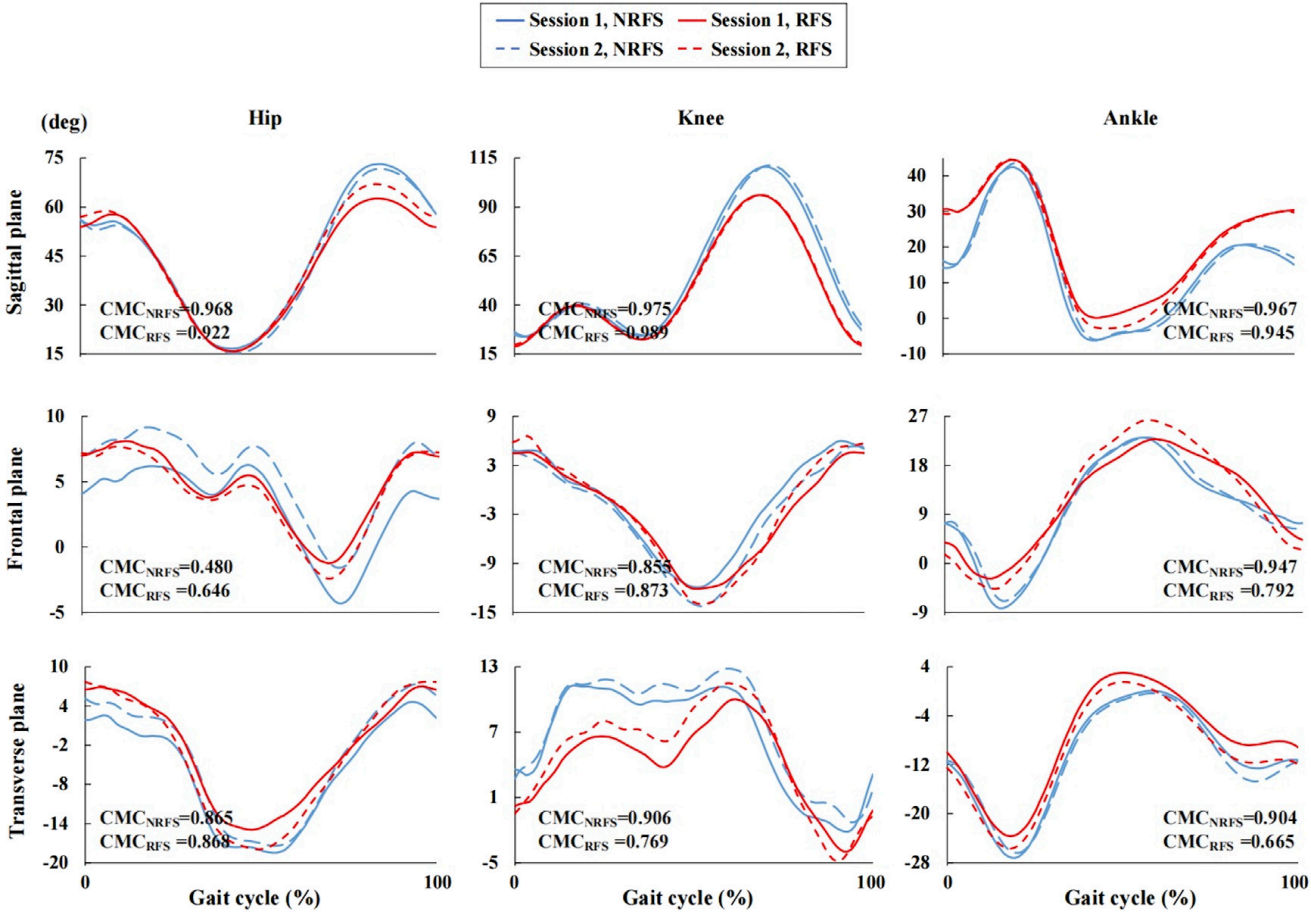
Joint angle waveforms during gait cycle measured by the inertial measurement units in session 1 and session 2.

**FIGURE 7:**
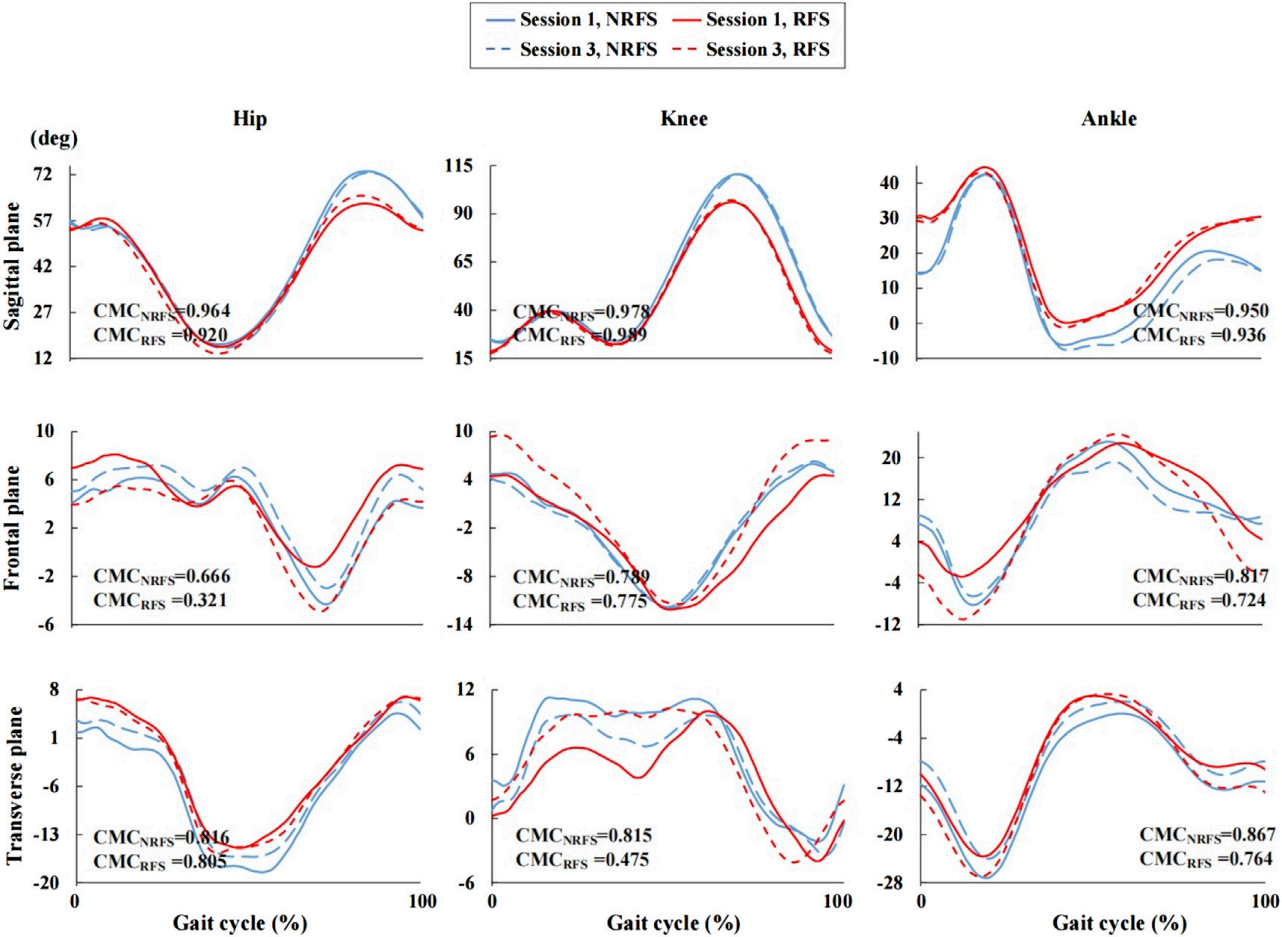
Joint angle waveforms during gait cycle measured by the inertial measurement units in session 1 and session 3. NRFS non-rearfoot strike pattern; RFS rearfoot strike pattern; CMC_NRFS_ the coefficient of multiple correlation in NRFS runners; CMC_RFS_ the coefficient of multiple correlation in RFS runners.

Under all conditions, the CMC of the kinematic waveforms in the sagittal plane showed very good to excellent performance (between-rater: NRFS: CMC = 0.967–0.975, RFS: CMC = 0.922–0.989; between-day: NRFS: CMC = 0.950–0.978, RFS: CMC = 0.920–0.989) in both patterns of runners (Figures 6, 7). In the frontal and transverse planes, for NRFS runners, the reliability of the joint angle waveforms was good to very good (between-rater: CMC = 0.855–0.947; between-day: CMC = 0.789–0.867), except for the hip joint angle waveforms in the frontal plane (between-rater: CMC = 0.480; between-day: CMC = 0.666); for RFS runners, the reliability of the joint angle waveforms was good to very good (between-rater: CMC = 0.769–0.873; between-day: CMC = 0.764–0.805), except for the hip joint angle waveforms in the frontal plane (between-rater: CMC = 0.646; between-day: CMC = 0.321), knee joint angle waveforms in the transverse plane (between-day: CMC = 0.475) and ankle joint angle waveforms in the frontal plane (between-day: CMC = 0.724) and transverse plane (between-rater: CMC = 0.665) (Figures 6, 7).

**FIGURE 8:**
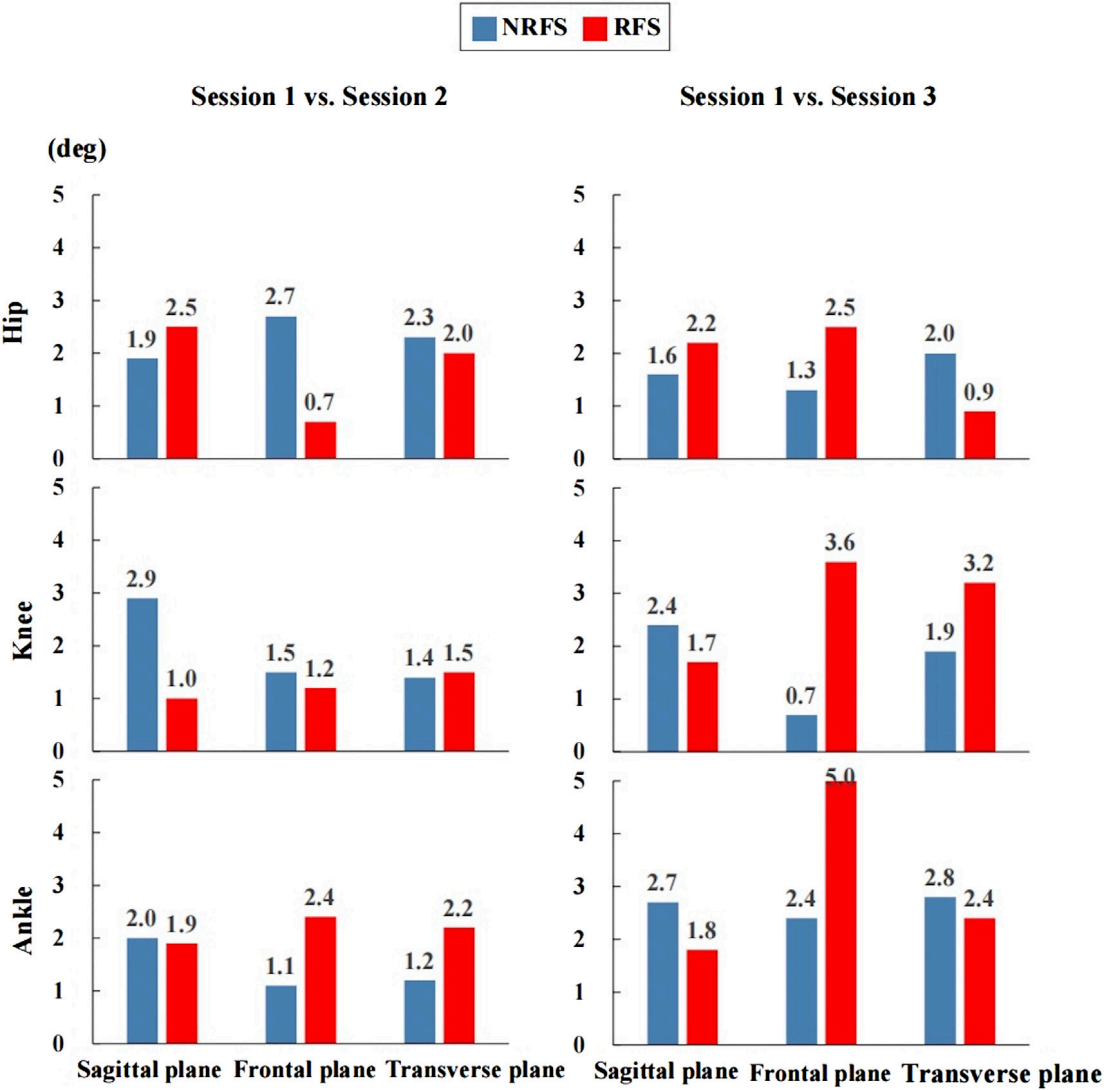
The root mean square error of the joint angle waveforms between the inertial measurement units system. NRFS non-rearfoot strike pattern; RFS rearfoot strike pattern.

The RMSEs of joint angle waveforms in the sagittal plane were in the ranges of 1.6°–2.9° (between-rater: RMSE = 1.9°–2.9°; between-day: RMSE = 1.6°–2.7°) and 1.0°–2.5° (between-rater: RMSE = 1.0°–2.5°; between-day: RMSE = 1.7°–2.2°) for all joints in NRFS and RFS runners, respectively. The RMSEs of joint angle waveforms in the frontal plane were in the ranges of 0.7°–2.7° (between-rater: RMSE = 1.1°–2.7°; between-day: RMSE = 0.7°–2.4°) and 0.7°–5.0° (between-rater: RMSE = 0.7°–2.4°; between-day: RMSE = 2.5°–5.0°) for all joints in NRFS and RFS runners, respectively. The RMSEs of joint angle waveforms in the transverse plane were in the ranges of 1.2°–2.8° (between-rater: RMSE = 1.2°–2.3°; between-day: RMSE = 1.9°–2.8°) and 0.9°–3.2° (between-rater: RMSE = 1.5°–2.2°; between-day: RMSE = 0.9°–3.2°) for all joints in NRFS and RFS runners, respectively (Figure 8).

### Discrete parameters

#### Correlation

For discrete parameters other than ROM, the between-rater reliability was moderate to excellent (Hip: ICC = 0.696–0.727; knee: ICC = 0.460–0.867; ankle: ICC = 0.645–0.938) in the sagittal plane, moderate to excellent (Hip: ICC = 0.479–0.814; knee: ICC = 0.735–0.788; ankle: ICC = 0.710–0.944) in the frontal plane and good to excellent (Hip: ICC = 0.539–0.783; knee: ICC = 0.764–0.882; ankle: ICC = 0.859–0.970) in the transverse plane in the NRFS runners; the between-day reliability was poor to excellent (Hip: ICC = 0.625–0.818; knee: ICC = 0.038–0.700; ankle: ICC = 0.194–0.920) in the sagittal plane, poor to good (Hip: ICC = 0.545–0.735; knee: ICC = 0.253–0.848; ankle: ICC = 0.194–0.826) in the frontal plane and poor to good (Hip: ICC = 0.514–0.741; knee: ICC = 0.338–0.764; ankle: ICC = 0.342–0.720) in the transverse plane in the RFS runners (Supplementary Table S2).

For the ROM parameters, the between-rater reliability was moderate to excellent (Hip: ICC = 0.566–0.742; knee: ICC = 0.798–0.930; ankle: ICC = 0.730–0.897) in the sagittal plane, poor to good (Hip: ICC = -0.034–0.392; knee: ICC = 0.739–0.828; ankle: ICC = 0.823–0.881) in the frontal plane and moderate to excellent (Hip: ICC = 0.749–0.776; knee: ICC = 0.729–0.967; ankle: ICC = 0.879–0.942) in the transverse plane in the NRFS runners; the between-day reliability was moderate to excellent (Hip: ICC = 0.789–0.915; knee: ICC = 0.726–0.789; ankle: ICC = 0.709–0.917) in the sagittal plane, poor to moderate (Hip: ICC = 0.510–0.645; knee: ICC = 0.217–0.460; ankle: ICC = 0.591–0.689) in the frontal plane and poor to good (Hip: ICC = 0.504–0.570; knee: ICC = 0.263–0.681; ankle: ICC = 0.694–0.746) in the transverse plane in the RFS runners (Supplementary Table S2).

For discrete parameters other than ROM, the between-day reliability was moderate to excellent (Hip: ICC = 0.445–0.713; knee: ICC = 0.624–0.921; ankle: ICC = 0.463–0.895) in the sagittal plane, moderate to good (Hip: ICC = 0.729–0.792; knee: ICC = 0.541–0.743; ankle: ICC = 0.606–0.893) in the frontal plane and moderate to excellent (Hip: ICC = 0.524–0.628; knee: ICC = 0.566–0.821; ankle: ICC = 0.829–0.933) in the transverse plane in the NRFS runners; the between-day reliability was poor to good (Hip: ICC = 0.732–0.866; knee: ICC = 0.235–0.791; ankle: ICC = 0.160–0.877) in the sagittal plane, poor to good (Hip: ICC = 0.257–0.747; knee: ICC = 0.161–0.659; ankle: ICC = -0.005–0.722) in the frontal plane and poor to good (Hip: ICC = 0.380–0.803; knee: ICC = -0.107–0.603; ankle: ICC = -0.018–0.789) in the transverse plane in the RFS runners (Supplementary Table S2).

For the ROM parameters, the between-day reliability was poor to good (Hip: ICC = 0.403–0.761; knee: ICC = 0.773–0.829; ankle: ICC = 0.182–0.681) in the sagittal plane, moderate to good (Hip: ICC = 0.632–0.700; knee: ICC = 0.637–0.778; ankle: ICC = 0.577–0.769) in the frontal plane and moderate to excellent (Hip: ICC = 0.573–0.779; knee: ICC = 0.516–0.934; ankle: ICC = 0.690–0.834) in the transverse plane in the NRFS runners; the between-day reliability was poor to good (Hip: ICC = 0.475–0.744; knee: ICC = 0.149–0.695; ankle: ICC = 0.437–0.622) in the sagittal plane, poor to moderate (Hip: ICC = 0.229–0.498; knee: ICC = 0.307–0.704; ankle: ICC = 0.340–0.374) in the frontal plane and poor to good (Hip: ICC = 0.436–0.885; knee: ICC = 0.641–0.705; ankle: ICC = 0.260–0.694) in the transverse plane in the RFS runners (Supplementary Table S2).

### RMSE

#### Hip

Supplementary Table S3 presents results from the RMSE of discrete parameters for the between-rater and between-day reliability. In the sagittal plane, the calculated RMSEs for the between-rater were in the ranges of 4.3°–7.6° and 2.5°–8.8° in NRFS and RFS runners, respectively; the calculated RMSEs for the between-day were in the ranges of 4.9°–8.0° and 4.6°–7.3° in NRFS and RFS runners, respectively. In the frontal plane, the calculated RMSEs for the between-rater were in the ranges of 4.5°–8.1° and 3.0°–6.0° in NRFS and RFS runners, respectively; the calculated RMSEs for the between-day were in the ranges of 3.8°–6.6° and 4.1°–9.3° in NRFS and RFS runners, respectively. In the transverse plane, the calculated RMSEs for the between-rater were in the ranges of 4.3°–7.5° and 5.0°–7.1° in NRFS and RFS runners, respectively; the calculated RMSEs for the between-day were in the ranges of 3.8°–9.7° and 2.6°–8.9° in NRFS and RFS runners, respectively.

#### Knee

Supplementary Table S3 presents results from the RMSE of discrete parameters for the between-rater and between-day reliability. In the sagittal plane, the calculated RMSEs for the between-rater were in the ranges of 1.9°–9.2° and 5.2°–16.4° in NRFS and RFS runners, respectively; the calculated RMSEs for the between-day were in the ranges of 3.0°–9.0° and 5.0°–21.7° in NRFS and RFS runners, respectively. In the frontal plane, the calculated RMSEs for the between-rater were in the ranges of 3.1°–6.4° and 3.4°–8.3° in NRFS and RFS runners, respectively; the calculated RMSEs for the between-day were in the ranges of 4.6°–8.1° and 4.1°–9.1° in NRFS and RFS runners, respectively. In the transverse plane, the calculated RMSEs for the between-rater were in the ranges of 3.5°–7.8° and 3.8°–6.3° in NRFS and RFS runners, respectively; the calculated RMSEs for the between-day were in the ranges of 5.4°–8.1° and 4.1°–9.6° in NRFS and RFS runners, respectively.

#### Ankle

Supplementary Table S3 presents results from the RMSE of discrete parameters for the between-rater and between-day reliability. In the sagittal plane, the calculated RMSEs for the between-rater were in the ranges of 2.9°–6.8° and 3.7°–15.9° in NRFS and RFS runners, respectively; the calculated RMSEs for the between-day were in the ranges of 4.1°–9.8° and 3.9°–18.1° in NRFS and RFS runners, respectively. In the frontal plane, the calculated RMSEs for the between-rater were in the ranges of 4.5°–8.3° and 6.1°–15.9° in NRFS and RFS runners, respectively; the calculated RMSEs for the between-day were in the ranges of 5.3°–13.1° and 6.3°–20.0° in NRFS and RFS runners, respectively. In the transverse plane, the calculated RMSEs for the between-rater were in the ranges of 4.9°–7.5° and 5.5°–13.8° in NRFS and RFS runners, respectively; the calculated RMSEs for the between-day were in the ranges of 5.0°–10.1° and 5.1°–17.1° in NRFS and RFS runners, respectively.

## Discussion

The aim aimed to assess the validity and reliability of the 3D joint kinematic outcomes between NRFS and RFS runners obtained by the IMUs system. To the authors’ knowledge, the current study was the first study to investigate the effect of running landing patterns on the validity and reliability of the IMUs joint kinematic measurement. Therefore, the results of this study could provide a theoretical basis for the application of IMUs in running performance monitoring. Results in the current study depicted that the measured sagittal kinematics of joint angle waveforms showed a high validity between the two systems after the offset correction. RFS runners generally had a higher validity than NRFS runners. The validity of the joint kinematics in the frontal and transverse planes measured by the IMUs system revealed high variability. In both types of runners, the joint kinematics in the sagittal plane showed very good to excellent performance in terms of between-rater and between-day reliability, whereas the reliability was weak to very good in the frontal and transverse planes across all joints. NRFS runners showed generally greater reliability than RFS runners.

### Validity

#### Waveforms

For the validity of the joint angle waveforms in the sagittal plane, our results showed very good correlation for the knee joint and only weak correlation for the hip and ankle joints before the offset correction. After the offset correction, the joint angle waveforms all showed very good correlations in two types of runners, which is similar to a previous study (Nuesch et al., 2017). Specifically, for the knee joint, the CMC of the joint angle waveform was generally higher in RFS runners than in NRFS runners, and the opposite was true for the hip joint in the sagittal plane before and after the offset was removed. The CMC of ankle joint was similar in the two types of runners. The variance in joint angle waveforms exhibited by runners with different FSPs can be explained by the fact that different impact sites during landing caused the IMUs to detect different impact accelerations (Napier et al., 2022). In addition, the accelerometer included in the STT system had an operating range of ± 16 g. Studies have shown that peak foot accelerations may exceed this operating range during running, which may affect the correct detection of foot strike events (Mitschke et al., 2018; van Werkhoven et al., 2019).

The results showed a more apparent offset in the sagittal plane for the hip and ankle joints than for the knee joint profile, which may be due to the relatively greater misalignment that occurs in the IMUs, which were fixed at the sacrum and the anterior upper shank during running. Similar to previous studies, our results also suggest that misalignment between IMUs may be the main cause of measurement errors (Bergmann et al., 2009; Ru et al., 2022). Compared to retroreflective markers that are usually affixed to the bone landmarks, IMUs positioned in the middle of the body segment which tend to have larger dimensions, making the IMUs more susceptible to soft tissue motion (Bergmann et al., 2009).

The CMCs of joint angle waveforms in the frontal and transverse planes were also investigated to explore the effect of FSPs on the IMUs measurements in a multidimensional context. Our study derived similar results to previous studies (Ferrari et al., 2010b; Roislien et al., 2012), showing that the CMC values of most joint angle waveforms was a complex number in both the frontal and transverse planes. This finding suggested a significant difference between the joint angle waveforms obtained by the two systems in the frontal and transverse planes. This may be attributed to the larger ROM in the sagittal plane during running, resulting in more kinematic cross-talk in other planes of motion (Lebleu et al., 2020; Kim et al., 2021).

In contrast to previous studies on slower activities (e.g., walking), our study showed lower correlations and larger RMSEs between the lower limb joint angle waveforms measured by the IMUs system and the optical motion capture system during running (Al-Amri et al., 2018; Patel et al., 2022). Similar results were obtained in some dynamic tasks (e.g., jumping and soccer athletic manoeuvres), which may indicate that more caution should be taken when applying the IMUs system to more dynamic sports (Akins et al., 2015; Al-Amri et al., 2018). The RMSE of all joints in the sagittal plane ranged from 18° to 28° before the offset was removed, and from 5° to 8° after the offset was removed when running at participants’ self-selected running speed (Nuesch et al., 2017). Both before and after the offset correction, our results showed that the RMSE of the joint waveform in NRFS runners (before: 16.1°–27.2°; after: 4.6°–13.7°) was comparable with those contained in a previous study, whereas the RMSE of the joint waveform in RFS runners (before: 9.1°–25.7°; after: 3.1°–7.7°) was smaller than that obtained in a previous study. Admittedly, the faster running speed may have contributed to the lower validity of lower extremity joint kinematics. Inevitably, both systems were subjected to soft tissue artefacts (Cutti et al., 2005; Forner-Cordero et al., 2008; Fasel et al., 2018; de Ruiter et al., 2022), and despite our efforts to secure the IMUs to the segment in various ways in the pilot study, such as using elastic tape and nylon straps and modifying the position of the IMUs prior to the start of formal trial, the movement of soft tissues due to the vigorous oscillations during running can lead to measurement errors (Nuesch et al., 2017; Wouda et al., 2018; Kim et al., 2021).

### Discrete parameters

To further investigate the characteristics of the running gait, discrete parameters were analysed in this study. The differences of ROM-related parameters in the sagittal plane between the two systems were also significantly smaller compared with other discrete parameters, indicating that the STT system was appropriate for ROM measurements in the sagittal plane, but the peak joint angles and the angle at a certain time point in the sagittal plane and parameters in other planes of motion measured by the STT system and the optical reference system were not interchangeable, which was consistent with findings of previous studies (Nuesch et al., 2017; Koska et al., 2018). As described above for the joint angle waveforms, there was a significant offset between the systems, resulting in large RMSE values between the discrete parameters. We speculated that the recognition error for foot strike and toe-off events is the main reason for the apparent offset generation. To optimise the IMUs measurements, several studies have redefined gait event detection methods using different data, such as peak downward velocity of the pelvis (Reenalda et al., 2021), minima/maxima of the acceleration (Schmidt et al., 2016; Mo and Chow, 2018; Donahue and Hahn, 2022), pitch angular velocity (Falbriard et al., 2018) and foot inclination angle in the sagittal plane (Nazarahari et al., 2022). However, to improve the generalisability of our results, the gait event identification results available automatically from the STT system were used, instead of manual identification.

Before the offset correction, discrete parameters in all cases, except for those of the ankle joint in the sagittal and frontal planes, generally showed higher RMSE in NRFS runners than in RFS runners. The parameters of the ankle joint in the sagittal and transverse planes were also depicted to have larger RMSE than those of the hip and knee joints. RFS runners presented a stiffer running gait pattern, i.e., less overall movement of the ankle joint, which may be responsible for the inconsistency (Kuhman et al., 2016; Yin et al., 2020). The results could imply that inaccurate estimation of gait events causes a greater impact on the determination of discrete parameters associated with the ankle joint (van Werkhoven et al., 2019). After the offset correction, the RMSE between the discrete parameters was comparable in both types of runners. Although the correlation of the discrete parameters between the two systems also increased significantly after the offset was removed, the correlations on the frontal plane (Hip: r = 0.136–0.854; knee: r = 0.139–0.796; ankle: r = -0.393–0.796) and transverse plane (Hip: r = 0.143–0.954; knee: r = 0.211–0.904; ankle: r = -0.154–0.796) remained poorer compared with that on the sagittal plane (Hip: r = 0.518–0.957; knee: r = 0.493–0.971; ankle: r = -0.150–0.965). The combination of higher variability and smaller ROM in the frontal and transverse planes during running resulted in a higher offset in the planes (Kim et al., 2021). Previous reviews have also suggested that care should be taken when using joint angles measured by the IMUs system in the frontal and transverse planes for clinical reasoning (van der Straaten et al., 2018; Poitras et al., 2019; Kobsar et al., 2020).

Previous studies have applied IMUs to identify the FSPs; van Werkhove et al. (van Werkhoven et al., 2019) obtained a recognition rate of 92.2%, whereas DeJong and Hertel (DeJong and Hertel, 2020) obtained a recognition rate of 78%. In our study, the foot touchdown angles differed significantly between the two systems, especially before the offset correction (before the offset correction: NRFS runner: 11.0°–29.6°; RFS runners: 2.6°–6.8°; after the offset correction: NRFS runner: 2.6°–6.8°; RFS runners: 3.2°–4.8°). The different methods/models used to calculate joint kinematics between the two systems may be the main reason for the discrepancy (Zhang et al., 2013; Mavor et al., 2020). The Vicon system calculated the angle of the joint based on the position of the retroreflective markers by the relative angle link, whereas the IMU system determined the joint angle by integrating the raw signals from the sensors (accelerometer, gyroscope and magnetometer) to identify the relative position change between the segments (Jaysrichai et al., 2015). In general, through the correlations and differences of the joint kinematics, the STT system provided a more accurate measure of lower extremity joint kinematics in RFS runners compared with NRFS runners. Thus, the results of the validity of the IMUs measurement in NRFS runners must be interpreted more cautiously.

### Reliability

#### Waveforms

Between-rater reliability and between-day reliability in the sagittal plane were shown to be very good to excellent performance for lower extremity joint angle waveforms of runners with different FSPs, which was comparable with the results in a systematic review of the reliability of three-dimensional gait analysis (McGinley et al., 2009). The good reliability reported in the sagittal plane confirmed that the STT system was quite robust for the measurement of joint kinematics in the sagittal plane of the lower extremity during running. However, the reliability was weak to very good (CMC = 0.321–0.947) across the joints in the frontal and transverse planes. NRFS runners (CMC = 0.480–0.947) showed generally higher reliability than RFS runners (CMC = 0.321–0.873). The RMSE between joint angle waveforms was less than 5° under all conditions, which was similar to the results previously obtained by the optical reference systems in gait analysis (McGinley et al., 2009; Meldrum et al., 2014).

In the frontal and transverse planes, NRFS runners’ joint angle waveforms showed generally higher reliability than those of RFS runners. The between-day reliability of the hip joint angle waveforms in the frontal plane (CMC = 0.321) and knee joint angle waveforms in the transverse plane (CMC = 0.475) of the RFS runners and between-rater reliability of the hip joint angle waveforms in the frontal plane (CMC = 0.480) of the NRFS runners showed the most variability. During the experiment, the asymmetry of muscle size on both sides of the spine and the bony prominence of the tibia tend to make the IMUs at the sacrum and at the anterior upper shank deviate to different degrees. Therefore, the misalignment of the IMUs at the sacrum and anterior upper shank may be the main reason for the contradictory results in the frontal and transverse planes (Bergmann et al., 2009). Overall, the between-day RMSEs were generally higher than the between-rater RMSEs, which may indicate that individual differences in participants’ running patterns had greater influence on the reliability of IMUs measurements than technical issues.

### Discrete parameters

The reliability of the discrete parameters was not as high as we expected. More specifically, knee and ankle joints-related parameters showed greater RMSE in the RFS runners, whereas hip joint-related parameters performed comparably in both types of runners. The difference in gait event recognition mentioned above may be the main reason. Thus, more caution is needed when interpreting discrete parameters measured by the IMU system. Although previous studies have suggested that calibration procedures affect the accuracy and reliability of the measurements (Bouvier et al., 2015; Lebleu et al., 2020), both systems were calibrated with participants in a neutral standing position in the current study. The difference between the measurements of the two systems due to calibration was therefore negligible.

### Limitations

Only one commercial inertial sensor system was validated in this study, which limited the generalisability of the results to some extent. The external synchronisation system used in the current study allowed the same gait to be analysed, but there may still exist a delay that resulted in an offset between the joint angle waveforms. The inevitable movement of soft tissues during running may also lead to misalignment of the IMUs. In addition, the gait events were determined by different identification methods in the two systems, and the resulting slight differences may be converted to differences in the foot strike time points between the two systems, which in turn affected the accuracy of the IMUs measurements.

## Conclusion

The study highlighted the possibility of using IMUs system for running kinematic measurements, but the accuracy of lower extremity joint kinematics varied depending on FSP, plane of movement and the joint/segment tracked. In conclusion, the IMUs system was an eligible tool for detecting changes in lower extremity joint kinematics in the sagittal plane during running, especially for RFS runners. However, RFS runners performed less reliability than NRFS runners. The joint kinematics data in frontal and transverse planes derived by the IMUs system need to be used with caution in clinical decision making. Researchers should consider variations in accuracy when discerning the clinical significance of joint kinematic changes captured by the IMUs. The IMU system can be used as an alternative tool for studying running gait patterns.

## Data Availability

The original contributions presented in the study are included in the article/Supplementary Material, further inquiries can be directed to the corresponding author.
