# Supplementary material for: Validity and reliability of inertial measurement units measurements for running kinematics in different foot strike pattern runners
Source: Front Bioeng Biotechnol. 2022 Dec 8;10:1005496. doi: 10.3389/fbioe.2022.1005496 (PMC9793257; doi:10.3389/fbioe.2022.1005496)
Supplement: Supplementary file 1 [file Table1.docx]

Supplementary Table S1. Spearman’s correlation coefficient and root mean square error of the discrete parameter.

|  | | | Before offset correction | | | | | | After offset correction | | | | | |
| --- | --- | --- | --- | --- | --- | --- | --- | --- | --- | --- | --- | --- | --- | --- |
|  |  |  | Sagittal plane | | Frontal plane | | Transverse plane | | Sagittal plane | | Frontal plane | | Transverse plane | |
|  |  |  | r | RMSE | r | RMSE | r | RMSE | r | RMSE | r | RMSE | r | RMSE |
| Hip | Touchdown angle | NRFS | -0.357 | 29.6° | 0.393 | 9.3° | -0.157 | 9.7° | 0.693* | 4.9° | 0.282 | 2.6° | 0.679* | 5.9° |
|  |  | RFS | 0.057 | 26.3° | -0.111 | 8.0° | 0.107 | 8.2° | **0.796*** | 3.2° | 0.471 | 3.0° | **0.896*** | 6.1° |
|  | Maximum angle in the cycle | NRFS | 0.339 | 31.5° | 0.264 | 7.9° | -0.207 | 9.6° | **0.946*** | 3.2° | 0.343 | 3.9° | **0.754*** | 6.7° |
|  |  | RFS | -0.043 | 29.4° | -0.007 | 7.1° | -0.075 | 8.3° | **0.761*** | 3.8° | 0.671* | 2.0° | **0.814*** | 6.4° |
|  | Maximum angle in the stance phase | NRFS | -0.243 | 31.2° | 0.500 | 7.9° | 0.000 | 9.0° | **0.811*** | 4.0° | 0.496 | 3.7° | **0.893*** | 5.2° |
|  |  | RFS | 0.118 | 30.6° | -0.093 | 7.5° | 0.043 | 7.8° | **0.900*** | 4.5° | 0.561* | 2.7° | **0.954*** | 5.7° |
|  | Maximum angle in the swing phase | NRFS | 0.296 | 31.5° | 0.150 | 7.5° | -0.164 | 9.6° | **0.957*** | 3.2° | 0.136 | 3.9° | 0.732* | 7.9° |
|  |  | RFS | 0.068 | 28.2° | 0.125 | 7.7° | 0.100 | 8.7° | **0.850*** | 3.3° | **0.854*** | 2.8° | **0.764*** | 6.9° |
|  | Minimum angle in the cycle | NRFS | 0.014 | 25.3° | -0.004 | 9.5° | -0.204 | 16.7° | **0.854*** | 4.0° | 0.343 | 4.2° | 0.411 | 9.0° |
|  |  | RFS | -0.032 | 24.8° | -0.125 | 6.7° | 0.318 | 12.8° | 0.707* | 4.7° | **0.761*** | 2.9° | **0.800*** | 8.8° |
|  | Minimum angle in the stance phase | NRFS | 0.250 | 23.2° | 0.175 | 9.0° | -0.579* | 17.3° | **0.850*** | 6.5° | 0.161 | 3.6° | 0.143 | 8.8° |
|  |  | RFS | 0.103 | 23.1° | 0.293 | 5.4° | 0.136 | 13.7° | **0.767*** | 6.6° | 0.575* | 2.4° | 0.579* | 8.8° |
|  | Minimum angle in the swing phase | NRFS | 0.004 | 25.7° | -0.004 | 9.5° | -0.204 | 16.6° | **0.871*** | 3.5° | 0.343 | 4.2° | 0.450 | 8.8° |
|  |  | RFS | -0.179 | 27.5° | -0.154 | 6.7° | 0.379 | 12.2° | 0.518* | 6.5° | **0.757*** | 2.9° | **0.807*** | 8.0° |
|  | ROM in the cycle | NRFS | **0.864*** | 7.0° | 0.061 | 7.8° | 0.300 | 15.2° | / | / | / | / | / | / |
|  |  | RFS | 0.686* | 7.6° | 0.376 | 4.1° | 0.175 | 14.9° | / | / | / | / | / | / |
|  | ROM in the stance phase | NRFS | 0.286 | 9.0° | -0.114 | 5.4° | 0.396 | 13.0° | / | / | / | / | / | / |
|  |  | RFS | 0.575* | 8.5° | 0.096 | 4.2° | 0.301 | 14.2° | / | / | / | / | / | / |
|  | ROM in the swing phase | NRFS | **0.861*** | 6.5° | -0.154 | 7.5° | 0.243 | 16.5° | / | / | / | / | / | / |
|  |  | RFS | 0.336 | 7.6° | -0.007 | 5.6° | 0.239 | 14.3° | / | / | / | / | / | / |
| Knee | Touchdown angle | NRFS | 0.479 | 18.0° | 0.229 | 7.6° | -0.239 | 18.4° | 0.600* | 6.8° | 0.525* | 9.7° | 0.354 | 8.8° |
|  |  | RFS | 0.345 | 11.0° | 0.343 | 5.9° | **0.789*** | 5.9° | **0.779*** | 4.2° | 0.139 | 8.6° | **0.904*** | 7.3° |
|  | Maximum angle in the cycle | NRFS | 0.404 | 12.0° | 0.114 | 8.2° | -0.114 | 24.4° | **0.971*** | 3.9° | 0.650* | 8.6° | 0.529* | 9.5° |
|  |  | RFS | 0.257 | 8.7° | 0.138 | 6.0° | -0.064 | 15.8° | **0.871*** | 3.1° | 0.301 | 7.0° | 0.407 | 6.4° |
|  | Maximum angle in the stance phase | NRFS | -0.107 | 13.5° | 0.200 | 7.8° | -0.211 | 22.4° | **0.904*** | 3.2° | 0.361 | 8.9° | 0.589* | 8.1° |
|  |  | RFS | 0.175 | 13.8° | 0.244 | 5.9° | 0.343 | 11.4° | 0.696* | 8.7° | 0.209 | 7.6° | **0.889*** | 2.4° |
|  | Maximum angle in the swing phase | NRFS | 0.404 | 12.0° | 0.204 | 8.3° | -0.043 | 24.8° | **0.971*** | 3.9° | **0.796*** | 8.2° | 0.536* | 9.8° |
|  |  | RFS | 0.275 | 8.1° | 0.292 | 5.6° | -0.261 | 16.3° | **0.882*** | 3.1° | 0.442 | 6.9° | 0.211 | 6.9° |
|  | Minimum angle in the cycle | NRFS | 0.639* | 14.8° | 0.029 | 15.1° | -0.079 | 9.0° | 0.711* | 4.4° | 0.611* | 8.6° | 0.614* | 14.0° |
|  |  | RFS | 0.398 | 11.7° | 0.746* | 13.4° | -0.054 | 7.4° | 0.736* | 5.2° | 0.632* | 9.5° | 0.511 | 7.6° |
|  | Minimum angle in the stance phase | NRFS | 0.289 | 12.7° | 0.086 | 10.6° | -0.261 | 15.7° | 0.493 | 3.2° | 0.675* | 5.2° | 0.218 | 8.5° |
|  |  | RFS | 0.248 | 10.1° | 0.436 | 7.8° | 0.404 | 5.9° | 0.723* | 3.2° | 0.175 | 3.7° | 0.689* | 8.5° |
|  | Minimum angle in the swing phase | NRFS | 0.629* | 17.9° | 0.039 | 15.1° | -0.075 | 9.8° | 0.696* | 7.0° | 0.611* | 8.6° | 0.604* | 14.0° |
|  |  | RFS | 0.459 | 12.8° | 0.682* | 13.1° | 0.111 | 7.9° | **0.754*** | 6.3° | 0.457 | 9.4° | 0.564* | 6.8° |
|  | ROM in the cycle | NRFS | **0.882*** | 7.7° | 0.039 | 17.0° | 0.000 | 23.2° | / | / | / | / | / | / |
|  |  | RFS | 0.707* | 9.2° | 0.130 | 16.2° | 0.090 | 12.6° | / | / | / | / | / | / |
|  | ROM in the stance phase | NRFS | 0.686* | 4.6° | -0.204 | 11.3° | 0.075 | 9.8° | / | / | / | / | / | / |
|  |  | RFS | 0.029 | 9.5° | -0.286 | 10.1° | 0.455 | 2.9° | / | / | / | / | / | / |
|  | ROM in the swing phase | NRFS | **0.829*** | 10.5° | 0.046 | 16.6° | 0.046 | 23.1° | / | / | / | / | / | / |
|  |  | RFS | 0.686* | 10.0° | 0.325 | 15.7° | 0.196 | 12.8° | / | / | / | / | / | / |
| Ankle | Touchdown angle | NRFS | 0.671* | 27.1° | 0.161 | 7.8° | 0.096 | 22.0° | **0.954*** | 2.6° | 0.168 | 11.1° | 0.068 | 8.2° |
|  |  | RFS | 0.350 | 29.2° | 0.011 | 10.7° | 0.304 | 14.3° | **0.807*** | 4.8° | 0.055 | 15.2° | **0.796*** | 4.4° |
|  | Maximum angle in the cycle | NRFS | 0.161 | 7.8° | 0.546* | 18.0° | 0.611* | 21.5° | 0.186 | 14.2° | 0.650* | 10.2° | 0.696* | 12.8° |
|  |  | RFS | 0.011 | 10.7° | -0.182 | 20.0° | 0.493 | 7.4° | 0.248 | 18.3° | 0.301 | 11.0° | 0.500 | 9.3° |
|  | Maximum angle in the stance phase | NRFS | 0.532* | 28.4° | 0.457 | 14.7° | 0.236 | 20.4° | **0.768*** | 4.8° | 0.361 | 6.9° | 0.421 | 5.7° |
|  |  | RFS | 0.473 | 30.1° | 0.042 | 15.6° | 0.282 | 10.5° | 0.692* | 5.0° | 0.209 | 6.6° | 0.600* | 6.7° |
|  | Maximum angle in the swing phase | NRFS | 0.589* | 27.4° | 0.486 | 17.8° | 0.611* | 21.5° | **0.943*** | 3.0° | **0.796*** | 10.2° | 0.696* | 12.8° |
|  |  | RFS | 0.556* | 27.6° | -0.182 | 20.0° | 0.504 | 7.5° | **0.965*** | 2.3° | 0.442 | 11.0° | 0.554* | 8.8° |
|  | Minimum angle in the cycle | NRFS | 0.579* | 24.2° | -0.036 | 12.1° | -0.450* | 29.4° | **0.871*** | 2.1° | -0.393 | 15.0° | 0.218 | 12.7° |
|  |  | RFS | **0.886*** | 24.0° | 0.186 | 8.5° | -0.125 | 24.6° | **0.957*** | 2.7° | 0.589* | 11.4° | 0.411 | 12.8° |
|  | Minimum angle in the stance phase | NRFS | -0.211 | 16.6° | -0.068 | 12.7° | -0.121 | 30.1° | -0.150 | 13.1° | -0.414 | 15.3° | -0.154 | 14.3° |
|  |  | RFS | 0.626* | 15.2° | 0.171 | 8.5° | -0.051 | 25.2° | 0.631* | 14.0° | 0.589* | 11.3° | 0.371 | 13.4° |
|  | Minimum angle in the swing phase | NRFS | 0.629* | 24.8° | 0.600* | 4.0° | -0.350 | 22.7° | **0.925*** | 1.9° | 0.118 | 7.7° | 0.646* | 3.9° |
|  |  | RFS | **0.850*** | 25.8° | 0.029 | 9.8° | 0.037 | 12.4° | **0.925*** | 3.3° | 0.504 | 11.5° | 0.574* | 3.1° |
|  | ROM in the cycle | NRFS | 0.636* | 6.3° | -0.318 | 24.8° | 0.414 | 25.0° | / | / | / | / | / | / |
|  |  | RFS | **0.854*** | 7.5° | 0.055 | 21.5° | 0.364 | 21.4° | / | / | / | / | / | / |
|  | ROM in the stance phase | NRFS | 0.068 | 16.5° | -0.471 | 20.4° | 0.307 | 17.3° | / | / | / | / | / | / |
|  |  | RFS | 0.604* | 17.0° | 0.305 | 16.6° | 0.305 | 19.4° | / | / | / | / | / | / |
|  | ROM in the swing phase | NRFS | **0.939*** | 3.9° | 0.175 | 16.9° | 0.486 | 15.7° | / | / | / | / | / | / |
|  |  | RFS | 0.693* | 5.7° | -0.414 | 22.7° | 0.367 | 10.5° | / | / | / | / | / | / |

NRFS non-rearfoot strike pattern; RFS rearfoot strike pattern; r spearman's correlation coefficient; RMSE root mean square error; ROM range of motion; * indicates the p-value is less than 0.05; bold indicates a strong correlation.
