# Supplementary material for: Validity and reliability of inertial measurement units measurements for running kinematics in different foot strike pattern runners
Source: Front Bioeng Biotechnol. 2022 Dec 8;10:1005496. doi: 10.3389/fbioe.2022.1005496 (PMC9793257; doi:10.3389/fbioe.2022.1005496)
Supplement: Supplementary file 2 [file Table2.docx]

Supplementary Table S2. The intra-class correlation coefficients with 95% confidence intervals of the discrete parameter measured by the inertial measurement units system.

|  | | | Session 1 vs. Session 2 | | | Session 1 vs. Session 3 | | |
| --- | --- | --- | --- | --- | --- | --- | --- | --- |
|  |  |  | Sagittal plane | Frontal plane | Transverse plane | Sagittal plane | Frontal plane | Transverse plane |
| Hip | Touchdown angle | NRFS | 0.704 (0.306, 0.890) | 0.674 (0.285, 0.876) | 0.630 (0.206, 0.857) | 0.677 (0.264, 0.879) | 0.770 (0.443, 0.916) | 0.500 (0.010, 0.798) |
|  |  | RFS | 0.813 (0.489, 0.936) | 0.735 (0.367, 0.903) | 0.741 (0.394, 0.904) | 0.765 (0.427, 0.915) | 0.345 (-0.151, 0.713) | 0.417 (-0.131, 0.761) |
|  | Maximum angle in the cycle | NRFS | 0.709 (0.333, 0.892) | 0.778 (0.446, 0.920) | 0.539 (0.091, 0.813) | 0.711 (0.326, 0.893) | 0.792 (0.500, 0.924) | 0.543 (0.079, 0.817) |
|  |  | RFS | 0.710 (0.327, 0.892) | 0.641 (0.196, 0.864) | 0.656 (0.221, 0.871) | 0.732 (0.368, 0.902) | 0.747 (0.412, 0.906) | 0.474 (-0.052, 0.789) |
|  | Maximum angle in the stance phase | NRFS | 0.696 (0.293, 0.887) | 0.786 (0.458, 0.924) | 0.579 (0.144, 0.832) | 0.682 (0.270, 0.881) | 0.787 (0.487, 0.922) | 0.386 (-0.124, 0.739) |
|  |  | RFS | 0.743 (0.393, 0.905) | 0.670 (0.245, 0.876) | 0.730 (0.358, 0.901) | 0.776 (0.464, 0.918) | 0.733 (0.378, 0.901) | 0.491 (-0.027, 0.796) |
|  | Maximum angle in the swing phase | NRFS | 0.710 (0.334, 0.892) | 0.814 (0.505, 0.935) | 0.563 (0.122, 0.825) | 0.713 (0.328, 0.894) | 0.776 (0.446, 0.919) | 0.628 (0.202, 0.857) |
|  |  | RFS | 0.815 (0.539, 0.933) | 0.721 (0.339, 0.898) | 0.607 (0.142, 0.850) | 0.759 (0.415, 0.913) | 0.717 (0.359, 0.894) | 0.380 (-0.175, 0.742) |
|  | Minimum angle in the cycle | NRFS | 0.726 (0.371, 0.898) | 0.609 (0.178, 0.848) | 0.750 (0.417, 0.907) | 0.473 (-0.043, 0.787) | 0.753 (0.406, 0.910) | 0.295 (-0.216, 0.687) |
|  |  | RFS | 0.818 (0.552, 0.934) | 0.612 (0.174, 0.850) | 0.549 (0.103, 0.818) | 0.866 (0.644, 0.953) | 0.419 (-0.050, 0.751) | 0.602 (0.132, 0.848) |
|  | Minimum angle in the stance phase | NRFS | 0.727 (0.361, 0.899) | 0.479 (0.011, 0.784) | 0.783 (0.473, 0.921) | 0.539 (0.043, 0.819) | 0.729 (0.357, 0.901) | 0.224 (-0.303, 0.648) |
|  |  | RFS | 0.625 (0.182, 0.857) | 0.545 (0.065, 0.820) | 0.697 (0.314, 0.886) | 0.805 (0.515, 0.930) | 0.257 (-0.284, 0.670) | 0.803 (0.520, 0.929) |
|  | Minimum angle in the swing phase | NRFS | 0.704 (0.332, 0.889) | 0.613 (0.185, 0.849) | 0.752 (0.421, 0.908) | 0.445 (-0.071, 0.772) | 0.752 (0.404, 0.909) | 0.301 (-0.208, 0.690) |
|  |  | RFS | 0.796 (0.506, 0.926) | 0.626 (0.196, 0.856) | 0.514 (0.054, 0.801) | 0.814 (0.528, 0.934) | 0.413 (-0.057, 0.747) | 0.527 (0.018, 0.814) |
|  | ROM in the cycle | NRFS | 0.742 (0.379, 0.906) | 0.392 (-0.161, 0.748) | 0.753 (0.423, 0.909) | 0.743 (0.380, 0.906) | 0.700 (0.319, 0.887) | 0.779 (0.455, 0.920) |
|  |  | RFS | 0.789 (0.488, 0.923) | 0.645 (0.198, 0.867) | 0.570 (0.109, 0.831) | 0.475 (-0.034, 0.787) | 0.318 (-0.156, 0.693) | 0.867 (0.658, 0.953) |
|  | ROM in the stance phase | NRFS | 0.566 (0.089, 0.830) | -0.034 (-0.575, 0.489) | 0.776 (0.455, 0.919) | 0.403 (-0.123,0.751) | 0.661 (0.259, 0.870) | 0.573 (0.087, 0.835) |
|  |  | RFS | 0.915 (0.766, 0.971) | 0.520 (0.040, 0.807) | 0.504 (0.032, 0.798) | 0.652 (0.227, 0.868) | 0.498 (-0.016, 0.799) | 0.436 (-0.099, 0.770) |
|  | ROM in the swing phase | NRFS | 0.718 (0.334, 0.896) | 0.391 (-0.163, 0.748) | 0.749 (0.416, 0.907) | 0.761 (0.418, 0.913) | 0.632 (0.220, 0.857) | 0.749 (0.403, 0.908) |
|  |  | RFS | 0.832 (0.566, 0.940) | 0.510 (0.056, 0.799) | 0.521 (0.060, 0.806) | 0.744 (0.396, 0.906) | 0.229 (-0.274, 0.645) | 0.885 (0.692, 0.960) |
| Knee | Touchdown angle | NRFS | 0.798 (0.502, 0.927) | 0.788 (0.471, 0.924) | 0.814 (0.542, 0.933) | 0.812 (0.523, 0.933) | 0.665 (0.240, 0.874) | 0.821 (0.545, 0.936) |
|  |  | RFS | 0.038 (-0.481, 0.529) | 0.780 (0.474, 0.919) | 0.764 (0.433, 0.914) | 0.237 (-0.273, 0.652) | 0.316 (-0.108, 0.681) | -0.093 (-0.603, 0.438) |
|  | Maximum angle in the cycle | NRFS | 0.468 (-0.064, 0.786) | 0.766 (0.448, 0.914) | 0.838 (0.588, 0.942) | 0.624 (0.170, 0.857) | 0.556 (0.061, 0.827) | 0.786 (0.473, 0.923) |
|  |  | RFS | 0.171 (-0.381, 0.622) | 0.834 (0.585, 0.940) | 0.349 (-0.192, 0.723) | 0.318 (-0.240, 0.709) | 0.322 (-0.105, 0.685) | 0.490 (0.000, 0.792) |
|  | Maximum angle in the stance phase | NRFS | 0.867 (0.655, 0.953) | 0.735 (0.377, 0.902) | 0.860 (0.636, 0.950) | 0.921 (0.783, 0.973) | 0.626 (0.180, 0.857) | 0.754 (0.411, 0.910) |
|  |  | RFS | 0.563 (0.107, 0.827) | 0.848 (0.608, 0.946) | 0.341 (-0.181, 0.716) | 0.235 (-0.262, 0.648) | 0.423 (-0.074, 0.761) | -0.107 (-0.550, 0.402) |
|  | Maximum angle in the swing phase | NRFS | 0.460 (-0.074, 0.782) | 0.769 (0.451, 0.915) | 0.846 (0.610, 0.945) | 0.627 (0.174, 0.858) | 0.541 (0.041, 0.820) | 0.759 (0.426, 0.912) |
|  |  | RFS | 0.178 (-0.374, 0.626) | 0.770 (0.454, 0.915) | 0.338 (-0.200, 0.717) | 0.311 (-0.248, 0.705) | 0.161 (-0.176, 0.547) | 0.479 (-0.015, 0.788) |
|  | Minimum angle in the cycle | NRFS | 0.829 (0.559, 0.939) | 0.769 (0.453, 0.915) | 0.796 (0.491, 0.927) | 0.901 (0.737, 0.965) | 0.737 (0.374, 0.903) | 0.566 (0.090, 0.830) |
|  |  | RFS | 0.646 (0.238, 0.863) | 0.311 (-0.250, 0.705) | 0.715 (0.352, 0.893) | 0.791 (0.489, 0.924) | 0.659 (0.252, 0.870) | 0.603 (0.139, 0.848) |
|  | Minimum angle in the stance phase | NRFS | 0.851 (0.609, 0.948) | 0.758 (0.423, 0.911) | 0.882 (0.683, 0.959) | 0.870 (0.665, 0.954) | 0.610 (0.155, 0.850) | 0.742 (0.404, 0.904) |
|  |  | RFS | 0.594 (0.155, 0.841) | 0.753 (0.424, 0.909) | 0.623 (0.174, 0.856) | 0.779 (0.473, 0.919) | 0.623 (0.191, 0.854) | 0.167 (-0.316, 0.602) |
|  | Minimum angle in the swing phase | NRFS | 0.686 (0.275, 0.883) | 0.773 (0.460, 0.917) | 0.764 (0.424, 0.915) | 0.844 (0.586, 0.945) | 0.743 (0.382, 0.906) | 0.615 (0.176, 0.851) |
|  |  | RFS | 0.700 (0.324, 0.887) | 0.253 (-0.308, 0.672) | 0.615 (0.154, 0.853) | 0.775 (0.456, 0.918) | 0.617 (0.187, 0.851) | 0.299 (-0.263, 0.699) |
|  | ROM in the cycle | NRFS | 0.829 (0.561, 0.939) | 0.739 (0.374, 0.905) | 0.967 (0.906, 0.989) | 0.788 (0.478, 0.923) | 0.777 (0.460, 0.919) | 0.931 (0.808, 0.976) |
|  |  | RFS | 0.736 (0.368, 0.903) | 0.285 (-0.255, 0.687) | 0.680 (0265, 0.881) | 0.695 (0.297, 0.886) | 0.686 (0.277, 0.883) | 0.705 (0.329, 0.889) |
|  | ROM in the stance phase | NRFS | 0.930 (0.705, 0.979) | 0.828 (0.558, 0.939) | 0.729 (0.375, 0.899) | 0.773 (0.335, 0.924) | 0.637 (0.190, 0.863) | 0.516 (0.046, 0.804) |
|  |  | RFS | 0.726 (0.366, 0.898) | 0.217 (-0.210, 0.618) | 0.263 (-0.298, 0.678) | 0.149 (-0.357, 0.597) | 0.307 (-0.114, 0.677) | 0.647 (0.206, 0.867) |
|  | ROM in the swing phase | NRFS | 0.798 (0.492, 0.928) | 0.796 (0.491, 0.927) | 0.965 (0.901, 0.988) | 0.829 (0.570, 0.939) | 0.778 (0.463, 0.919) | 0.934 (0.815, 0.977) |
|  |  | RFS | 0.789 (0.474, 0.924) | 0.460 (-0.024, 0.776) | 0.681 (0.291, 0.879) | 0.688 (0.288, 0.884) | 0.704 (0.255, 0.895) | 0.641 (0.233, 0.861) |
| Ankle | Touchdown angle | NRFS | 0.921 (0.738, 0.975) | 0.866 (0.645, 0.953) | 0.897 (0.719, 0.964) | 0.895 (0.715, 0.963) | 0.810 (0.537, 0.931) | 0.829 (0.547, 0.940) |
|  |  | RFS | 0.732 (0.372, 0.901) | 0.194 (-0.365, 0.637) | 0.426 (-0.097, 0.763) | 0.815 (0.544, 0.933) | 0.160 (-0.323, 0.598) | 0.458 (-0.033, 0.776) |
|  | Maximum angle in the cycle | NRFS | 0.866 (0.645, 0.953) | 0.939 (0.829, 0.979) | 0.967 (0.907, 0.989) | 0.810 (0.537, 0.931) | 0.648 (0.239, 0.865) | 0.919 (0.777, 0.972) |
|  |  | RFS | 0.194 (-0.365, 0.637) | 0.821 (0.545, 0.936) | 0.665 (0.269, 0.872) | 0.160 (-0.323, 0.598) | 0.722 (0.342, 0.898) | -0.018 (-0.174, 0.270) |
|  | Maximum angle in the stance phase | NRFS | 0.938 (0.828, 0.979) | 0.921 (0.780, 0.973) | 0.919 (0.778, 0.972) | 0.871 (0.658, 0.955) | 0.606 (0.166, 0.847) | 0.933 (0.817, 0.977) |
|  |  | RFS | 0.837 (0.581, 0.942) | 0.768 (0.435, 0.916) | 0.720 (0.351, 0.896) | 0.696 (0.307, 0.886) | 0.709 (0.339, 0.891) | 0.644 (0.206, 0.865) |
|  | Maximum angle in the swing phase | NRFS | 0.865 (0.649, 0.953) | 0.944 (0.842, 0.981) | 0.970 (0.916, 0.990) | 0.827 (0.572, 0.938) | 0.656 (0.250, 0.869) | 0.918 (0.774, 0.972) |
|  |  | RFS | 0.724 (0.371, 0.897) | 0.826 (0.560, 0.938) | 0.645 (0.236, 0.863) | 0.877 (0.647, 0.958) | 0.714 (0.327, 0.895) | 0.789 (0.480, 0.924) |
|  | Minimum angle in the cycle | NRFS | 0.891 (0.708, 0.962) | 0.857 (0.629, 0.949) | 0.861 (0.635, 0.951) | 0.763 (0.443, 0.913) | 0.893 (0.720, 0.963) | 0.843 (0.593, 0.945) |
|  |  | RFS | 0.920 (0.785, 0.972) | 0.699 (0.314, 0.887) | 0.418 (-0.114, 0.759) | 0.843 (0.603, 0.944) | -0.005 (-0.371, 0.441) | 0.261 (-0.272, 0.671) |
|  | Minimum angle in the stance phase | NRFS | 0.645 (0.239, 0.863) | 0.857 (0.628, 0.950) | 0.859 (0.631, 0.950) | 0.463 (-0.048, 0.781) | 0.873 (0.672, 0.955) | 0.844 (0.595, 0.945) |
|  |  | RFS | 0.832 (0.578, 0.940) | 0.717 (0.345, 0.895) | 0.411 (-0.122, 0.756) | 0.827 (0.562, 0.938) | 0.270 (-0.175, 0.657) | 0.257 (-0.275, 0.669) |
|  | Minimum angle in the swing phase | NRFS | 0.897 (0.723, 0.964) | 0.710 (0.318, 0.893) | 0.887 (0.695, 0.961) | 0.780 (0.476, 0.920) | 0.663 (0.236, 0.874) | 0.865 (0.559, 0.956) |
|  |  | RFS | 0.902 (0.740, 0.966) | 0.546 (0.057, 0.822) | 0.342 (-0.186, 0.718) | 0.873 (0.668, 0.955) | 0.295 (-0.168, 0.676) | 0.319 (-0.167, 0.696) |
|  | ROM in the cycle | NRFS | 0.840 (0.596, 0.943) | 0.875 (0.671, 0.956) | 0.942 (0.840, 0.980) | 0.604 (0.178, 0.844) | 0.687 (0.306, 0.881) | 0.803 (0.520, 0.929) |
|  |  | RFS | 0.919 (0.782, 0.972) | 0.689 (0.310, 0.882) | 0.720 (0.339, 0.897) | 0.552 (0.109, 0.820) | 0.373 (-0.102, 0.726) | 0.317 (-0.184, 0.698) |
|  | ROM in the stance phase | NRFS | 0.730 (0.370, 0.900) | 0.823 (0.549, 0.937) | 0.879 (0.686, 0.957) | 0.182 (-0.371, 0.628) | 0.577 (0.139, 0.832) | 0.834 (0.572, 0.941) |
|  |  | RFS | 0.809 (0.524, 0.931) | 0.591 (0.156, 0.838) | 0.746 (0.387, 0.907) | 0.437 (-0.084, 0.768) | 0.340 (-0.110, 0.701) | 0.694 (0.319, 0.884) |
|  | ROM in the swing phase | NRFS | 0.897 (0.713, 0.964) | 0.881 (0.683, 0.959) | 0.924 (0.792, 0.974) | 0.681 (0.276, 0.880) | 0.769 (0.446, 0.916) | 0.690 (0.311, 0.883) |
|  |  | RFS | 0.709 (0.318, 0.893) | 0.606 (0.169, 0.847) | 0.694 (0.289, 0.887) | 0.622 (0.204, 0.853) | 0.374 (-0.113, 0.728) | 0.260 (-0.201, 0.655) |

NRFS non-rearfoot strike pattern; RFS rearfoot strike pattern; ROM range of motion.
