# Supplementary material for: Validity and reliability of inertial measurement units measurements for running kinematics in different foot strike pattern runners
Source: Front Bioeng Biotechnol. 2022 Dec 8;10:1005496. doi: 10.3389/fbioe.2022.1005496 (PMC9793257; doi:10.3389/fbioe.2022.1005496)
Supplement: Supplementary file 3 [file Table3.docx]

Supplementary Table S3. The root mean square error of the discrete parameter measured by the inertial measurement units system.

|  | | | Session 1 vs. Session 2 | | | Session 1 vs. Session 3 | | |
| --- | --- | --- | --- | --- | --- | --- | --- | --- |
|  |  |  | Sagittal plane | Frontal plane | Transverse plane | Sagittal plane | Frontal plane | Transverse plane |
| Hip | Touchdown angle | NRFS | 7.1° | 7.5° | 7.0° | 7.2° | 6.6° | 7.8° |
|  |  | RFS | 6.2° | 5.8° | 5.3° | 6.8° | 9.3° | 8.6° |
|  | Maximum angle in the cycle | NRFS | 7.6° | 5.3° | 7.5° | 8.0° | 5.2° | 7.7° |
|  |  | RFS | 7.5° | 5.9° | 6.1° | 6.9° | 4.3° | 8.0° |
|  | Maximum angle in the stance phase | NRFS | 6.6° | 5.3° | 6.7° | 6.4° | 5.4° | 8.3° |
|  |  | RFS | 7.8° | 5.8° | 5.5° | 7.3° | 4.6° | 7.9° |
|  | Maximum angle in the swing phase | NRFS | 7.6° | 4.5° | 7.5° | 8.0° | 5.2° | 7.0° |
|  |  | RFS | 6.3° | 4.9° | 6.9° | 6.8° | 4.3° | 8.9° |
|  | Minimum angle in the cycle | NRFS | 5.3° | 7.8° | 5.0° | 6.6° | 6.1° | 8.0° |
|  |  | RFS | 6.4° | 6.1° | 6.8° | 5.7° | 7.6° | 6.6° |
|  | Minimum angle in the stance phase | NRFS | 4.8° | 8.1° | 5.6° | 5.6° | 5.3° | 9.7° |
|  |  | RFS | 8.8° | 4.5° | 6.8° | 6.8° | 6.8° | 5.2° |
|  | Minimum angle in the swing phase | NRFS | 5.4° | 7.8° | 5.0° | 6.6° | 6.1° | 8.0° |
|  |  | RFS | 7.3° | 6.0° | 7.1° | 6.9° | 7.6° | 7.4° |
|  | ROM in the cycle | NRFS | 4.3° | 5.5° | 4.3° | 5.1° | 4.2° | 3.8° |
|  |  | RFS | 4.2° | 3.0° | 5.0° | 5.9° | 4.5° | 2.6° |
|  | ROM in the stance phase | NRFS | 4.7° | 5.4° | 4.4° | 5.4° | 3.8° | 5.1° |
|  |  | RFS | 2.5° | 4.0° | 6.1° | 4.7° | 4.1° | 4.9° |
|  | ROM in the swing phase | NRFS | 4.5° | 5.3° | 4.5° | 4.9° | 4.7° | 4.4° |
|  |  | RFS | 4.2° | 3.3° | 5.5° | 4.6° | 5.0° | 2.6° |
| Knee | Touchdown angle | NRFS | 8.2° | 5.3° | 7.8° | 8.7° | 6.9° | 7.4° |
|  |  | RFS | 5.2° | 4.0° | 3.8° | 5.0° | 7.5° | 9.6° |
|  | Maximum angle in the cycle | NRFS | 9.1° | 5.7° | 6.7° | 9.0° | 7.8° | 7.4° |
|  |  | RFS | 12.6° | 3.4° | 5.8° | 12.1° | 7.9° | 7.1° |
|  | Maximum angle in the stance phase | NRFS | 5.3° | 5.9° | 6.4° | 4.2° | 7.2° | 8.1° |
|  |  | RFS | 16.4° | 3.5° | 5.0° | 21.7° | 7.2° | 9.4° |
|  | Maximum angle in the swing phase | NRFS | 9.2° | 5.8° | 6.5° | 8.9° | 8.1° | 7.9° |
|  |  | RFS | 12.6° | 4.1° | 6.3° | 12.2° | 9.1° | 7.8° |
|  | Minimum angle in the cycle | NRFS | 5.7° | 6.4° | 5.3° | 4.7° | 6.8° | 7.8° |
|  |  | RFS | 7.4° | 6.8° | 4.1° | 5.7° | 5.9° | 5.7° |
|  | Minimum angle in the stance phase | NRFS | 5.2° | 4.7° | 5.9° | 5.2° | 5.9° | 7.9° |
|  |  | RFS | 7.7° | 4.3° | 4.7° | 5.8° | 4.1° | 8.9° |
|  | Minimum angle in the swing phase | NRFS | 8.3° | 6.4° | 6.1° | 6.2° | 7.0° | 7.8° |
|  |  | RFS | 7.2° | 7.0° | 5.1° | 6.1° | 6.6° | 7.9° |
|  | ROM in the cycle | NRFS | 5.3° | 5.7° | 3.7° | 6.9° | 5.1° | 5.4° |
|  |  | RFS | 7.0° | 8.3° | 4.8° | 8.3° | 7.1° | 5.3° |
|  | ROM in the stance phase | NRFS | 1.9° | 3.1° | 3.5° | 3.0° | 4.6° | 4.6° |
|  |  | RFS | 9.7° | 5.7° | 5.8° | 17.8° | 5.6° | 4.1° |
|  | ROM in the swing phase | NRFS | 5.2° | 5.0° | 3.8° | 6.1° | 5.2° | 5.2° |
|  |  | RFS | 6.6° | 7.5° | 5.2° | 8.5° | 7.3° | 6.4° |
| Ankle | Touchdown angle | NRFS | 3.5° | 4.5° | 6.3° | 4.1° | 5.3° | 8.4° |
|  |  | RFS | 6.4° | 15.9° | 13.8° | 5.3° | 18.1° | 12.7° |
|  | Maximum angle in the cycle | NRFS | 4.5° | 5.1° | 5.6° | 5.3° | 11.8° | 8.5° |
|  |  | RFS | 15.9° | 6.1° | 8.1° | 18.1° | 6.9° | 17.1° |
|  | Maximum angle in the stance phase | NRFS | 3.2° | 4.5° | 6.8° | 4.3° | 9.5° | 6.1° |
|  |  | RFS | 4.0° | 6.3° | 8.2° | 5.9° | 6.3° | 8.3° |
|  | Maximum angle in the swing phase | NRFS | 4.5° | 5.0° | 5.4° | 5.0° | 11.8° | 8.6° |
|  |  | RFS | 6.0° | 6.1° | 8.4° | 3.9° | 7.1° | 5.1° |
|  | Minimum angle in the cycle | NRFS | 4.3° | 6.5° | 7.4° | 6.7° | 5.6° | 7.7° |
|  |  | RFS | 4.9° | 9.1° | 12.3° | 7.2° | 14.2° | 12.1° |
|  | Minimum angle in the stance phase | NRFS | 6.8° | 6.7° | 7.5° | 8.9° | 6.4° | 7.7° |
|  |  | RFS | 6.6° | 8.6° | 12.3° | 6.6° | 14.2° | 12.1° |
|  | Minimum angle in the swing phase | NRFS | 4.4° | 5.2° | 7.1° | 6.8° | 5.5° | 7.8° |
|  |  | RFS | 5.5° | 11.5° | 11.8° | 6.5° | 15.5° | 10.6° |
|  | ROM in the cycle | NRFS | 4.2° | 8.3° | 6.0° | 6.6° | 13.1° | 10.1° |
|  |  | RFS | 3.7° | 14.0° | 7.1° | 10.3° | 19.0° | 11.1° |
|  | ROM in the stance phase | NRFS | 5.9° | 7.3° | 4.9° | 9.8° | 12.2° | 5.0° |
|  |  | RFS | 5.9° | 13.5° | 5.5° | 9.9° | 17.0° | 5.9° |
|  | ROM in the swing phase | NRFS | 2.9° | 6.2° | 5.3° | 5.6° | 8.4° | 9.6° |
|  |  | RFS | 6.5° | 15.9° | 6.6° | 7.5° | 20.0° | 10.6° |

NRFS non-rearfoot strike pattern; RFS rearfoot strike pattern; ROM range of motion.
